# Rod-Like Nanoporous CeO_2_ Modified by PdO Nanoparticles for CO Oxidation and Methane Combustion with High Catalytic Activity and Water Resistance

**DOI:** 10.1186/s11671-019-3029-4

**Published:** 2019-06-06

**Authors:** Dong Duan, Chunxi Hao, Liqun Wang, Wenyu Shi, Haiyang Wang, Gege He, Lumei Gao, Zhanbo Sun

**Affiliations:** 1 School of Science, MOE Key Laboratory for Non-Equilibrium Synthesis and Modulation of Condensed Matter, Xi’an Jiaotong University, Xi’an, 710049 People’s Republic of China; 2 State Key Laboratory for Mechanical Behavior of Materials, Xi’an Jiaotong University, Xi’an, 710049 People’s Republic of China; 3 Key Laboratory of Shaanxi for Advanced Functional Materials and Mesoscopic Physics, Xi’an Jiaotong University, Xi’an, 710049 People’s Republic of China

**Keywords:** Al-Ce-Pd alloy ribbons, Dealloying, Nanoporous PdO/CeO_2_, CO oxidation, CH_4_ combustion

## Abstract

A PdO/CeO_2_ composite with a rod-like nanoporous skeletal structure was prepared by combining the dealloying of Al-Ce-Pd alloy ribbons with calcination. For CO oxidation and CH_4_ combustion, the nanoporous PdO/CeO_2_ composite exhibits excellent catalytic activity, and the complete reaction temperatures of CO and CH_4_ are 80 °C and 380 °C, respectively. In addition, the composite possesses excellent cycle stability, CO_2_ toxicity, and water resistance, and the catalytic activity hardly decreases after 100 h of long-term stability testing in the presence of water vapour (2 × 10^5^ ppm). The results of a series of characterizations indicate that the enhanced catalytic activity can be attributed to the good dispersion of the PdO nanoparticles, large specific surface area, strong redox capacity, interaction between PdO and CeO_2_, and more surface active oxygen on PdO. The results of the characterization and experiments also indicate that the PdO nanoparticles, prepared by combining dealloying and calcination, have a stronger catalytic activity than do Pd nanoparticles. Finally, a simple model is used to summarize the catalytic mechanism of the PdO/CeO_2_ composite. It is hoped that this work will provide insights into the development of high-activity catalysts.

**Electronic supplementary material:**

The online version of this article (10.1186/s11671-019-3029-4) contains supplementary material, which is available to authorized users.

## Background

At present, an increasing number of people are paying attention to environmental issues and focusing on mitigating several important environmental issues, such as exhaust emissions and global warming [1, 2]. In particular, the elimination of toxic CO and the greenhouse gas CH_4_ is the focus of a plethora of research. Among such investigations, low-temperature catalysis has proven to be an effective way to eliminate these polluting gases [3–6].

Although many studies have proven that cheap metals and their metal oxides (e.g. transition metals and oxides, and rare earth metals and oxides) can be used as catalysts for CO oxidation and CH_4_ combustion, it is undeniable that the use of noble metals usually significantly improves the catalytic performance [7, 8]. In recent years, Pd and PdO catalysts have been extensively studied and are considered to be some of the most effective catalysts for CO oxidation and CH_4_ combustion. They exhibit not only low volatility at high temperatures but also high catalytic activity at low temperatures [9, 10].

However, from an application point of view, since the abundance of precious metals in the earth is relatively low, the Pd and PdO catalysts that are generally used in practical industrial applications are loaded onto supports such as metal oxides, zeolites, carbon materials, and metal-organic frameworks. This configuration is also in line with the trend of developing sustainable catalysis by conserving noble metals and using the support-noble metal interaction to improve the catalytic activity [11, 12]. Among the types of supports, CeO_2_ is considered to be a promising support due to its strong oxygen storage/release properties and excellent thermal stability. For example, MacLachlan et al. used a combination of incipient wetness impregnation and surface-assisted reduction to prepare a nanostructured PdO/CeO_2_ composite, which exhibited a good activity as a catalyst for methane combustion after calcination [13]. Luo et al. reported the preparation of a PdO-CeO_2_ catalyst by a solution combustion method and proved that the synergistic effects of PdO and CeO_2_ are the reason for the enhanced catalytic activity [14].

Although many good results have been achieved, there are still some challenges. For example, many organic chemicals or surfactants may contaminate the nanomaterials, resulting in an insufficient catalytic activity, which is common with wet chemistry [15]. Furthermore, the process of preparing a catalyst based on the method of liquid precursor ageing is complicated, and the yield is low [16]. Therefore, the development of non-polluting, high-yield, and high catalytic activity materials remains a challenge.

In this work, we developed a method for the preparation of PdO/CeO_2_ composites by dealloying Al-Ce-Pd alloy ribbons and then calcinating. The preparation method is simple, the structure of the material is easy to control, and no organic reagents are needed [17, 18], which is particularly suitable for large-scale industrial production and a sustainable future [19, 20]. However, as far as we know, there have been no literature reports on the use of dealloying to prepare the catalysts for methane combustion. Therefore, it is hoped that this work can provide insights into and help with the synthesis and preparation of nanomaterials.

## Methods

### Materials

All chemicals and metals were used as received of analytical grade without further purification. Pure Al (99.90 wt%), pure Ce (99.90 wt%), and pure Pd (99.90 wt%) were from Sino-Platinum Metals Co., Ltd. Granular NaOH (AR) was from Shanghai Aladdin Biochemical Technology Co., Ltd. High-purity argon was from Xi’an Jiahe Co., Ltd.

### Synthesis of PdO/CeO_2_ Composite

Al_92−*X*_Ce_8_Pd_*X*_ (*X* = 0, 0.1, 0.3, 0.5, 0.7, 0.9, and 1.1) precursor alloys were prepared by arc melting pure Al, pure Ce, and pure Pd under an argon atmosphere. An obtained Al-Ce-Pd precursor alloy was re-melted by high-frequency induction heating in a quartz tube under argon protection. The molten alloy was blown onto a high-speed rotating copper roll by argon for rapid solidification, and an Al-Ce-Pd alloy ribbon with a width of approximately 3 to 4 mm and a thickness of approximately 20 to 30 μm was obtained.

**Fig. 1:**
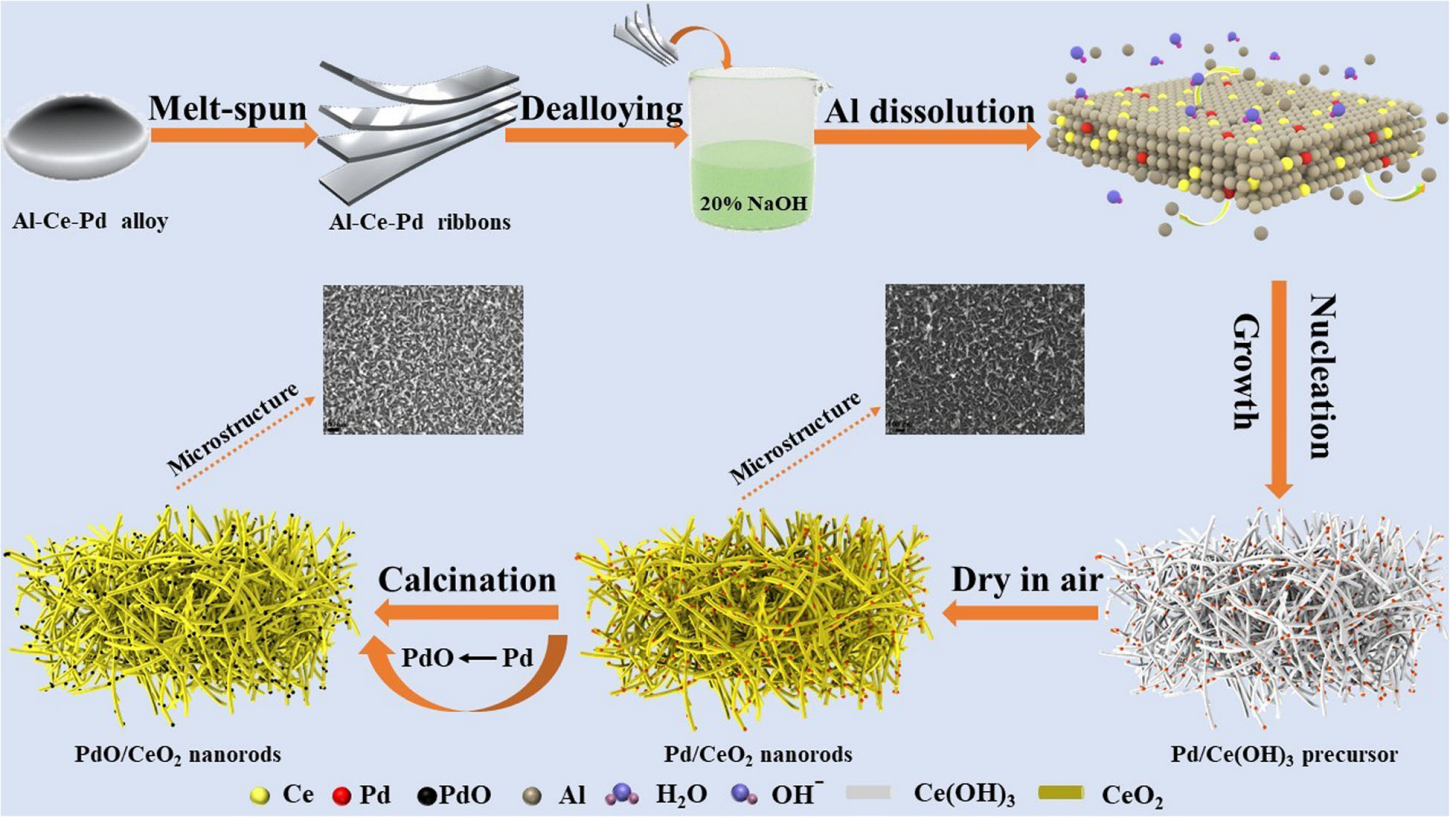
Schematic for the preparation of the rod-like nanoporous PdO/CeO_2_ composite

The prepared Al-Ce-Pd alloy ribbon was placed in a 20 wt% sodium hydroxide solution and dealloyed at ambient temperature for 2 h and then heated to 80 °C and dealloyed for 10 h. The dealloyed sample was repeatedly rinsed with deionized water and dried in air at room temperature. The dried sample was calcined in O_2_ at 200~600 °C for 2 h, and the flow rate of O_2_ is 18 mL min^−1^. The preparation method and structural evolution of the PdO/CeO_2_ composite are shown in Fig. 1.

### Sample Characterization

X-ray diffraction (XRD) patterns were obtained using a Shimadzu XRD-6100 diffractometer with Cu Kα radiation (20 kV and 40 mA). The morphology and elemental composition of the samples were obtained from a JSM-7000F scanning electron microscope (SEM) and an INCA X-Sight Oxford energy dispersive spectrometer (EDS). Transmission electron microscope (TEM) images, high-resolution transmission electron microscope (HRTEM) images, and scanning transmission electron microscopy images coupled with energy-dispersive X-ray spectra (STEM-EDX) were recorded on a JEOL JEM-200 electron microscope. The specific surface area (*S*_BET_), pore size (*D*_p_), and pore volume (*V*_p_) of the samples were determined with a Micromeritics ASAP 2020 apparatus at 77.4 K. The X-ray photoelectron spectroscopy (XPS) analysis was performed with a multifunctional spectrometer model Axis Ultra Kratos. Hydrogen temperature-programmed reduction (H_2_-TPR) was carried out on a Quantachrome Autosorb-iQC-TPX, in which a 50-mg sample was heated from 50 to 800 °C at a ramp rate of 10 °C/min in 10 vol% H_2_/Ar mixture gas flowing at a velocity of 40 mL min^−1^.

### Catalyst Testing

The catalytic activity of the samples was evaluated by CO oxidation and CH_4_ combustion. The catalyst (100 mg) was charged into a stainless steel tubular reactor, and the reactor was placed in a tube furnace with precise temperature control. A feed gas composed of either 1 vol% CO, 10 vol% O_2_, and 89 vol% N_2_ or 1 vol% CH_4_, 10 vol% O_2_, and 89 vol% N_2_ was introduced into the reactor at a total flow rate of 50 mL min^−1^ (space velocity = 30000 h^−1^), and the flow rate of the reaction gas was controlled and adjusted by a mass flow meter (Brooks 5850E). Details on the preparation of the feed gas with 20 vol% H_2_O are shown in the Additional file 1: Figure S1. The CO and CH_4_ concentrations during the heating process were analysed in-line by an Agilent GC-7890B gas chromatograph equipped with a flame ionization detector (FID). The conversion of CO and CH_4_ was calculated according to Eq. (1):

1 $$ X=\frac{C_{\mathrm{in}}-{C}_{\mathrm{out}}}{C_{\mathrm{in}}}\times 100\% $$

where *X* represents the conversion of CO or CH_4_, *C*_in_ represents the inlet concentration of CO or CH_4_, and *C*_out_ represents the outlet concentration of CO or CH_4_.

The reaction rates of CO and CH_4_ were calculated using Eq. (2) [14, 21]:

2 $$ {r}_{\mathrm{CO}/{\mathrm{CH}}_4}=\frac{C_{\mathrm{CO}/{\mathrm{CH}}_4}\cdot {X}_{\mathrm{CO}/{\mathrm{CH}}_4}\cdot P\cdot V}{m_{\mathrm{cat}}\cdot {W}_{\mathrm{Pd}}\cdot R\cdot T}\left(\mathrm{mol}\cdot {\mathrm{s}}^{-1}\cdot {\mathrm{g}}_{\mathrm{Pd}}^{-1}\right) $$

where *r*_CO/CH4_ represents the reaction rate of CO or CH_4_; the concentration of CO or CH_4_ is expressed as *C*_CO/CH4_ in the feed gas; the conversion of CO or CH_4_ is expressed as *X*_CO/CH4_; *P* is the atmospheric pressure, which is 101.3 KPa; *V* is the total flow rate; *m*_cat_ is the mass of the catalyst in the reactor; *W*_Pd_ is the loading of Pd; *R* is the molar gas constant, which is 8.314 Pa m^3^ mol^−1^ K^−1^; and *T* is the ambient temperature (293 K).

## Results and Discussion

### Catalyst Characterization

**Fig. 2:**
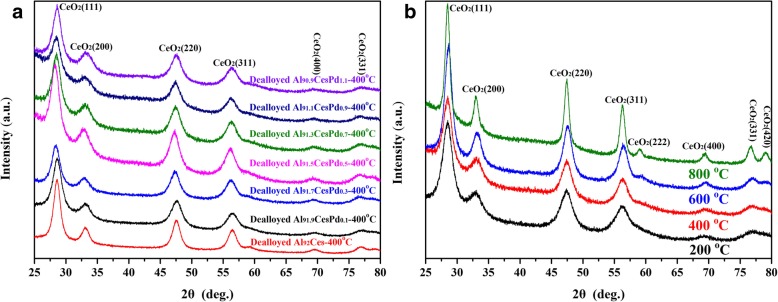
XRD patterns of Al_92−*X*_Ce_8_Pd_*X*_ (*X* = 0.1, 0.3, 0.5, 0.7, 0.9, and 1.1) dealloyed at 400 °C (**a**) and the XRD patterns of dealloyed Al_91.3_Ce_8_Pd_0.7_ alloys calcined at different temperatures (**b**)

The XRD spectra of the samples are shown in Fig. 2. A clear set of diffraction peaks for CeO_2_ (JCPDS NO. 34-0394) was detected, as shown in Fig. 2a, but the diffraction peaks associated with Pd were not detected in all dealloyed samples that were calcined at 400 °C. The dealloyed Al_91.3_Ce_8_Pd_0.7_ alloy was calcined at different temperatures, as shown in Fig. 2b. As the temperature gradually increased, the diffraction peaks of CeO_2_ gradually became sharp and narrow; however, even after an 800 °C calcination, the diffraction peaks related to Pd could not be detected. This result may be due to the relatively low concentration of Pd in the sample, which may be highly dispersed in the sample in the form of either an amorphous phase or small crystalline grains. No diffraction peaks associated with Al were observed in the XRD spectra for any of the samples, indicating that the residual Al content in the samples after dealloying was either very low or that Al was present in the samples in an amorphous form.

**Fig. 3:**
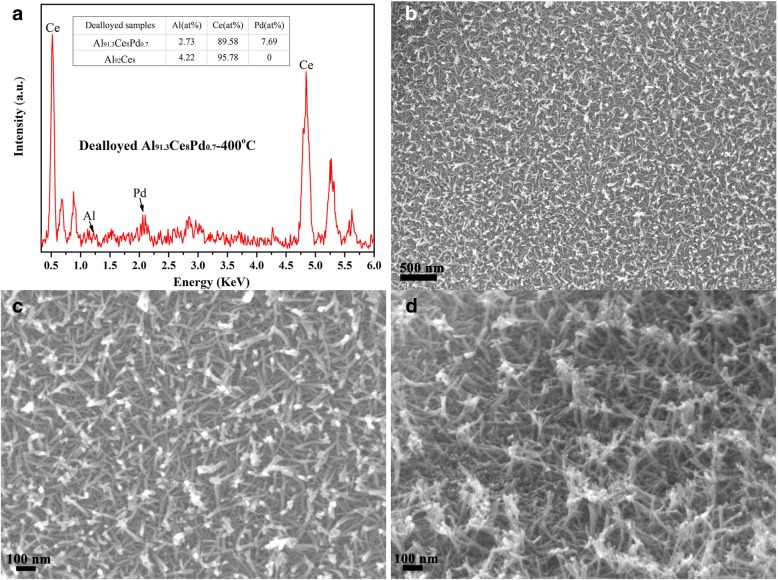
EDS pattern (**a**), surface SEM images (**b**, **c**), and cross-sectional SEM image (**d**) of the dealloyed Al_91.3_Ce_8_Pd_0.7_ alloy calcined at 400 °C

The EDS results and microstructures of the dealloyed Al_91.3_Ce_8_Pd_0.7_ calcined at 400 °C are shown in Fig. 3. After dealloying, the contents of Ce and Pd in the sample were very close to those in the precursor alloy, as shown in Fig. 3a, indicating that the loss of Ce and Pd was small during the dealloying process. The residual Al in the dealloyed Al_91.3_Ce_8_Pd_0.7_ alloy calcined at 400 °C was very low, only 2.73%, which is consistent with the XRD results. Figures 3b and c show the surface SEM images of the sample, and the sample exhibits a uniform, disordered, woven porous structure composed of numerous nanorods with a diameter of approximately 10 nm; the nanorods are stacked on top of each other to form several micropores and mesopores. A cross-sectional SEM image of the sample is shown in Fig. 3d, and the nanorods are connected to each other to form a three-dimensional skeletal structure that facilitates the stabilization of the nanoparticles through a large contact area, resulting in more nanoscale interactive interfaces.

**Fig. 4:**
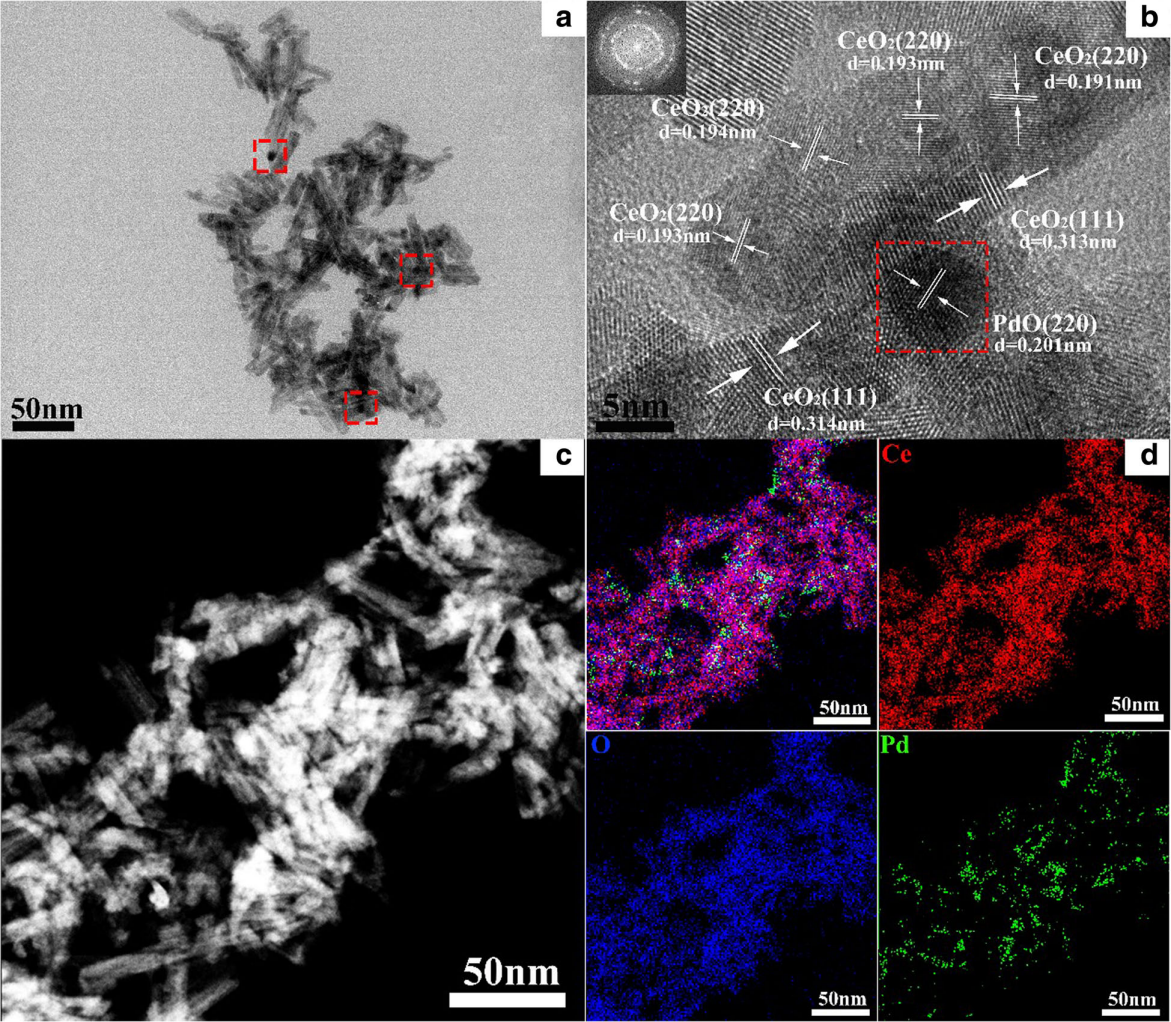
TEM (**a**), HRTEM (**b**), and TEM-EDS mapping (**c**, **d**) images of the dealloyed Al_91.3_Ce_8_Pd_0.7_ alloy calcined at 400 °C

TEM images and TEM-EDX mapping were further used to characterize the structures and particle distribution of the sample, as shown in Fig. 4. The dealloyed Al_91.3_Ce_8_Pd_0.7_ calcined at 400 °C consists of numerous nanorods, as shown in Fig. 4a, and the nanorods are stacked on each other to form many micropores and mesopores, which is consistent with the SEM results. There are several darker particles distributed on the surface of the nanorods, as shown in the red boxes in Fig. 4a. Figure 4b shows the HRTEM image of the sample. The lattice spacing values of the nanorods are approximately 0.314 nm and 0.193 nm, corresponding to the (111) and (220) planes of CeO_2_, respectively. The red box in Fig. 4b is the HRTEM image of the darker particles seen in Fig. 4a, which possess a diameter of approximately 5 nm and are distributed on the surface of the CeO_2_ nanorods. However, the lattice spacing value of the darker particles (0.201 nm) is similar to that of the (220) plane of CeO_2_ (0.193 nm) and is difficult to distinguish. The TEM-EDX mapping characterization was carried out to identify these particles. As shown in Figs. 4c and d, the particles distributed on the CeO_2_ nanorods are related to Pd, and considering the lattice spacing value of 0.201 nm, the darker particles are determined to be PdO nanoparticles that are uniformly dispersed on the surface of the CeO_2_ nanorods (Fig. 4d), forming a number of rough interfaces.

**Fig. 5:**
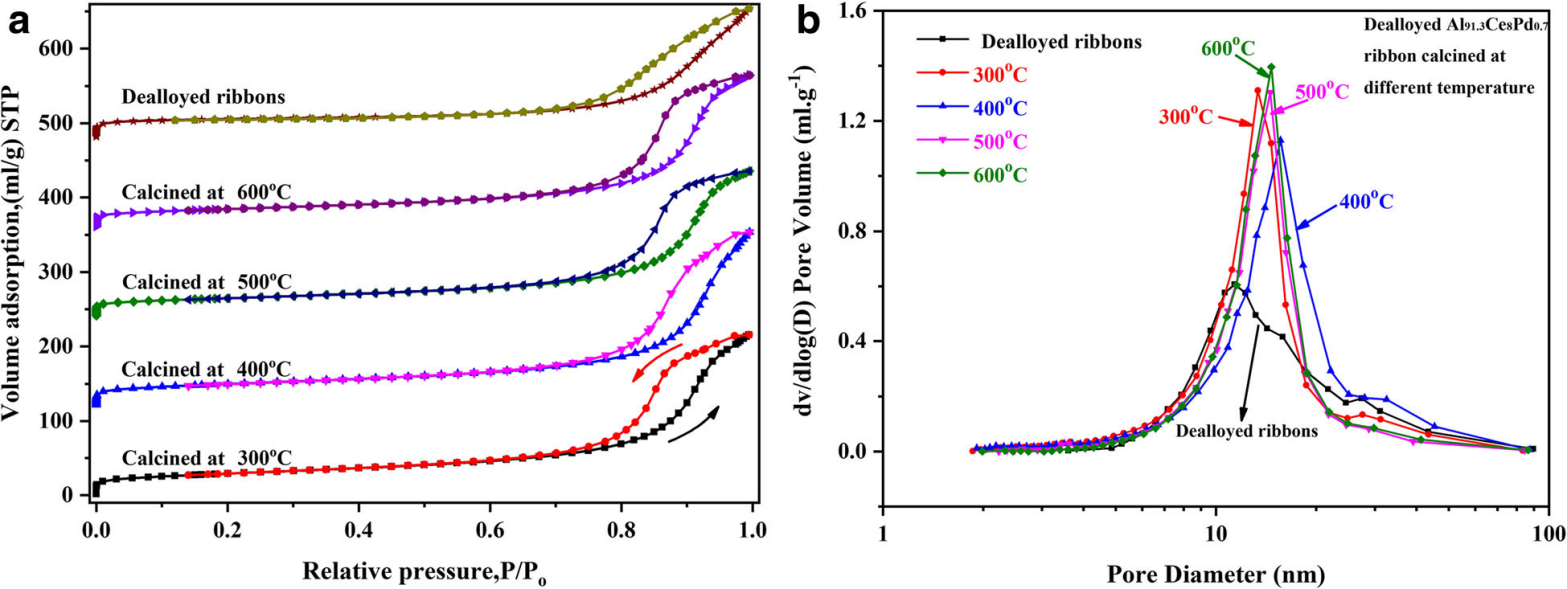
Nitrogen adsorption-desorption isotherm curves (**a**) and pore size distribution curves (**b**) of dealloyed Al_91.3_Ce_8_Pd_0.7_ ribbons calcined at different temperatures

The BET and BJH tests were performed on the dealloyed Al_91.3_Ce_8_Pd_0.7_ calcined at different temperatures, and the corresponding results are shown in Fig. 5 and Table 1. All samples exhibited type IV isotherms with H_2_ and H_3_ hysteresis loops, as shown in Fig. 5a. This result indicates that the samples are mesoporous structures, which is consistent with the SEM and TEM results. In addition, the pore size distribution curve shown in Fig. 5b further proves that all of the samples possess a mesoporous structure, and the calcined samples exhibited similarly narrow pore size distributions with an average pore diameter of approximately 12~14 nm. The specific surface area (*S*_BET_), pore size (*D*_p_), and pore volume (*V*_p_) of the samples at the corresponding calcination temperatures are listed in Table 1. The 400 °C calcined sample demonstrates the largest specific surface area and the largest pore volume equal to approximately 102 m^2^ g^−1^ and 0.362 cm^3^ g^−1^, respectively. As the calcination temperature was continuously increased, the specific surface area slightly decreased; however, even after the 600 °C calcination, the sample still had a specific surface area of 84 m^2^ g^−1^. These results indicate that the rod-like nanoporous CeO_2_ skeletal structure has an excellent anti-sintering ability. This conclusion is also verified by the crystalline sizes in Additional file 1: Table S1 and the SEM and TEM images in Additional file 1: Figure S2.

**Table 1:** Specific surface area (*S*_BET_), pore size (*D*_p_) and pore volume (*V*_p_) of the dealloyed Al_91.3_Ce_8_Pd_0.7_ ribbons calcined at different temperatures

Calcination temperature (°C)	*S*_BET_ (m^2^ g^−1^)	*D*_p_ (nm)	*V*_p_ (cm^3^ g^−1^)
Dealloyed ribbons	80	13.66	0.267
Calcined at 300 °C	101	12.02	0.336
Calcined at 400 °C	102	13.72	0.362
Calcined at 500 °C	85	12.73	0.304
Calcined at 600 °C	84	13.21	0.317

**Fig. 6:**
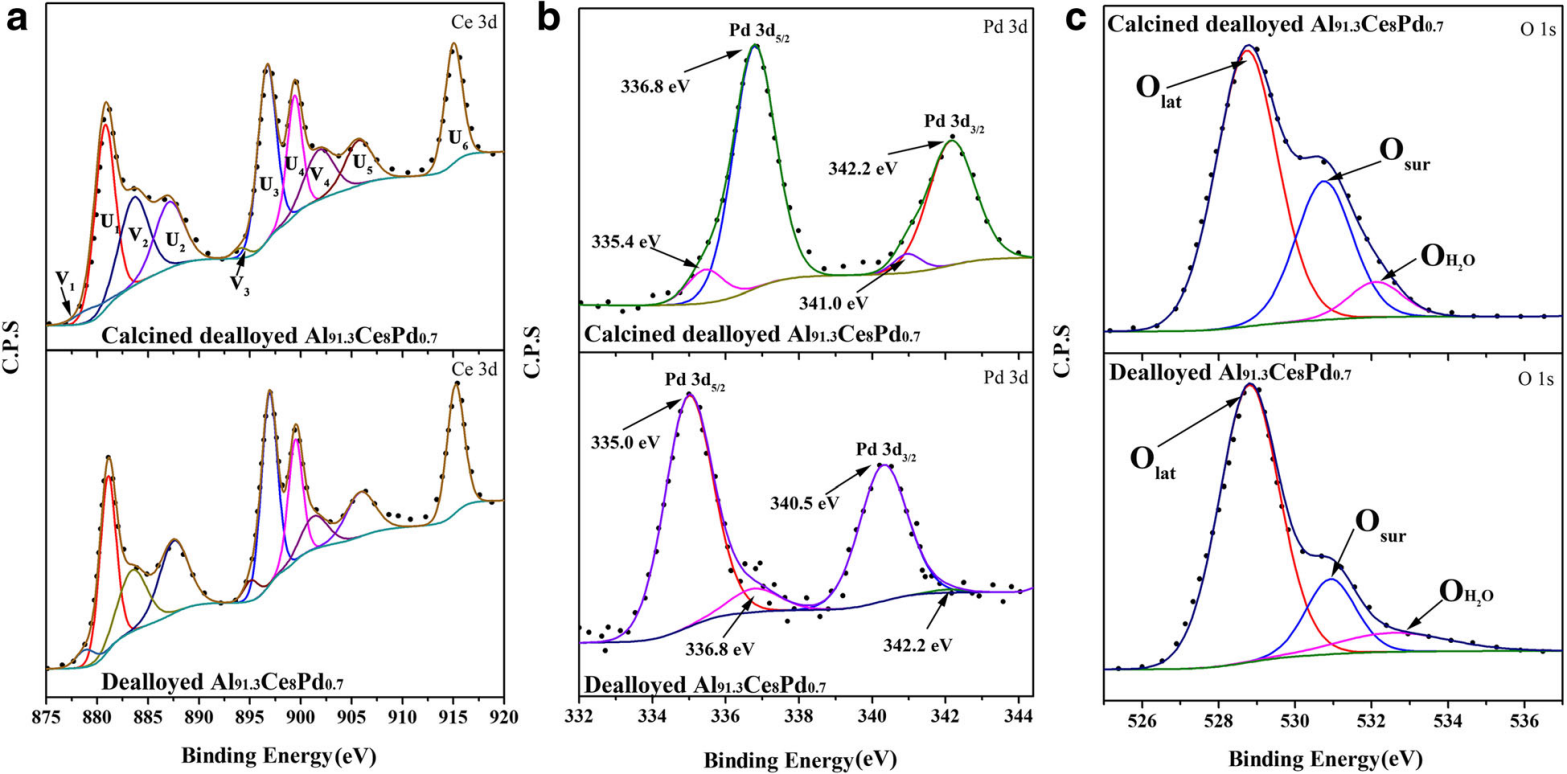
XPS spectra of the Ce 3d (**a**), Pd 3d (**b**), and O 1s (**c**) regions of the dealloyed Al_91.3_Ce_8_Pd_0.7_ sample and the dealloyed Al_91.3_Ce_8_Pd_0.7_ sample calcined at 400 °C

The XPS spectra of the dealloyed Al_91.3_Ce_8_Pd_0.7_ sample and the calcined dealloyed Al_91.3_Ce_8_Pd_0.7_ sample are shown in Fig. 6 to further characterize the valence composition of the surface elements of the samples. The XPS spectrum of Ce 3d is shown in Fig. 6a, where U represents Ce^4+^ and V represents Ce^3+^ [22]. Generally, the existence of Ce^3+^ is closely related to oxygen vacancies. The calculated results indicate that the concentrations of Ce^3+^ in the dealloyed Al_91.3_Ce_8_Pd_0.7_ sample and the calcined dealloyed Al_91.3_Ce_8_Pd_0.7_ sample were 21.15% and 23.33%, respectively, having little difference, as shown in Table 2. This outcome indicates that the calcination hardly affects the concentration of the surface oxygen vacancies. To understand the effect of PdO loading on the oxygen vacancy concentration of the sample, the XPS spectrum of the dealloyed Al_92_Ce_8_ sample calcined at 400 °C (pure CeO_2_) is shown in Additional file 1: Figure S3. The Ce^3+^ concentration was calculated to be approximately 14.27%, which is far less than that of the sample loaded with PdO. This phenomenon indicates that there is an interaction between PdO and CeO_2_ and that the presence of PdO can change the valence state of CeO_2_ surface elements and increase the concentration of surface oxygen vacancies.

**Table 2:** Ratios of Ce, Pd, and O in different states for different catalysts, as obtained from the XPS results

Catalysts	Ce^3+^/(Ce^3+^ + Ce^4+^) (%)	Pd^2+^/(Pd^0^ + Pd^2+^) (%)	O_sur_/(O_lat_ + O_sur_ + O_H2O_) (%)
Calcined dealloyed Al_92_Ce_8_	14.27	/	/
Calcined dealloyed Al_91.3_Ce_8_Pd_0.7_	23.33	91.25	29.3
Dealloyed Al_91.3_Ce_8_Pd_0.7_	21.15	6.45	16.2

To further investigate the effects of calcination on the surface PdO nanoparticles, the Pd 3d XPS spectra of the dealloyed Al_91.3_Ce_8_Pd_0.7_ sample and the calcined dealloyed Al_91.3_Ce_8_Pd_0.7_ sample are shown in Fig. 6b. There are two forms of Pd in the calcined dealloyed Al_91.3_Ce_8_Pd_0.7_ ribbon; the strong peaks at 336.8 eV and 342.2 eV can be attributed to PdO (Pd^2+^) [23], and the weak peaks at 335.4 eV and 341.0 eV can be attributed to metallic Pd (Pd^0^) [24]. Table 2 shows that the concentrations of Pd^2+^ and Pd^0^ were approximately 91.25% and 8.75%, respectively. However, the analysis results for the dealloyed Al_91.3_Ce_8_Pd_0.7_ sample are the opposite, and the concentrations of Pd^2+^ and Pd^0^ were approximately 6.45% and 93.55%, respectively. This finding indicates that Pd is present in the form of metallic Pd in the dealloyed sample, whereas after calcination, Pd was oxidized into PdO and uniformly dispersed on the surface of CeO_2_, which is consistent with the results of the HRTEM images.

It is well known that surface active oxygen (O_sur_) is usually an active oxygen species for catalytic reactions. Figure 6c shows the O 1s XPS spectra of the two catalysts. For the calcined dealloyed Al_91.3_Ce_8_Pd_0.7_ sample, the peaks at 528.9 eV, 530.6 eV, and 532.1 eV correspond to the lattice oxygen (O_lat_), surface active oxygen (O_sur_), and weakly adsorbed H_2_O (O_H2O_), respectively [25, 26]. The ratio of O_sur_ (O_sur_/(O_lat_ + O_sur_ + O_H2O_)) was calculated and is listed in Table 2. The ratios of O_sur_ for the dealloyed Al_91.3_Ce_8_Pd_0.7_ sample and the calcined dealloyed Al_91.3_Ce_8_Pd_0.7_ sample were approximately 16.2% and 29.3%, respectively, indicating the presence of more surface active oxygen species in the calcined dealloyed Al_91.3_Ce_8_Pd_0.7_ sample. The concentrations of Ce^3+^ in these two catalysts were similar (Fig. 6a), suggesting that PdO has a stronger ability to adsorb and activate O_2_ than do the metallic Pd nanoparticles. A separate experiment (Additional file 1) was designed to eliminate the interference of thermal activation on the experimental results, as shown in Additional file 1: Figure S4, and the results obtained also support the above conclusion.

**Fig. 7:**
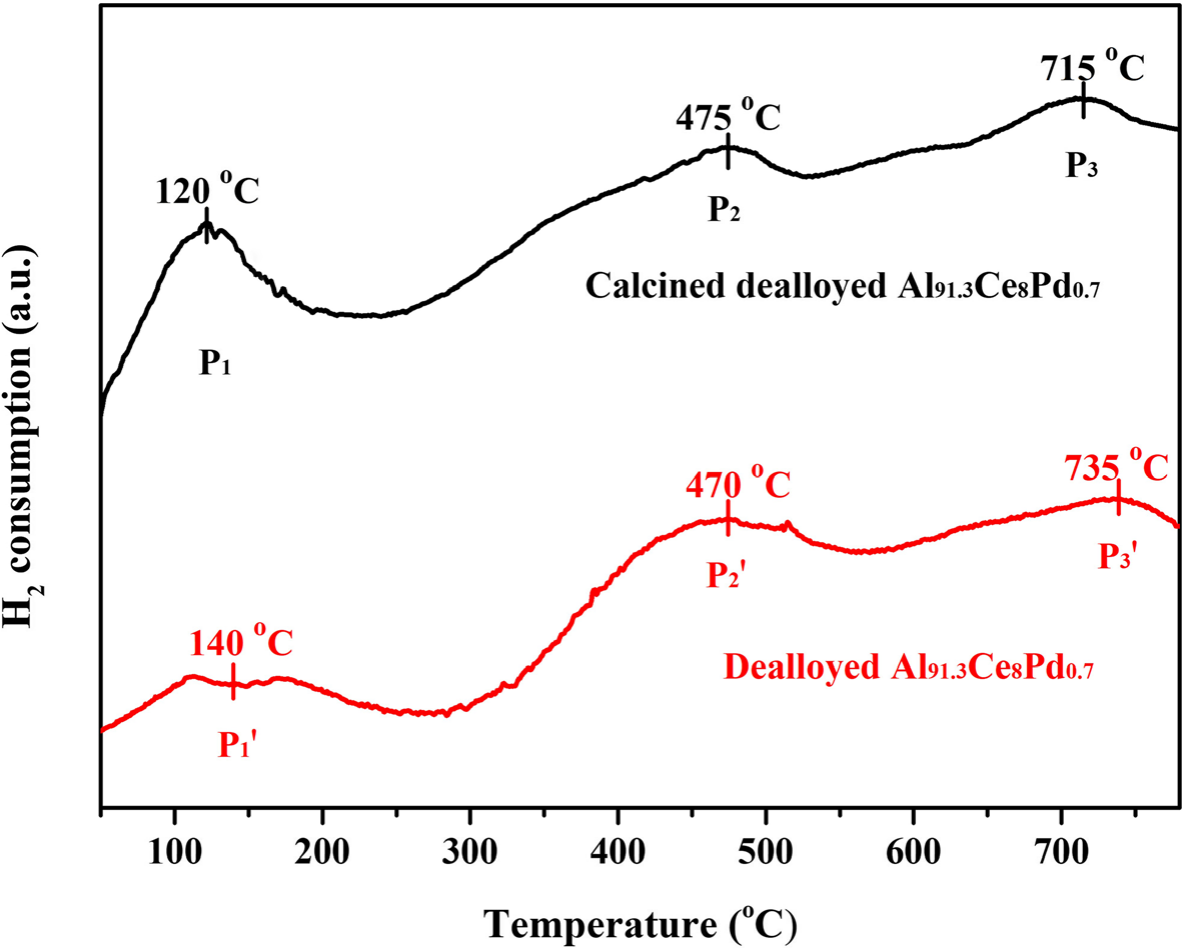
H_2_-TPR curves of the dealloyed Al_91.3_Ce_8_Pd_0.7_ ribbons and the dealloyed Al_91.3_Ce_8_Pd_0.7_ ribbons calcined at 400 °C

To compare the differences in the reducibility of the samples before and after calcination, H_2_-TPR was used to test both the calcined dealloyed Al_91.3_Ce_8_Pd_0.7_ sample and the dealloyed Al_91.3_Ce_8_Pd_0.7_ sample, and the results are shown in Fig. 7. There are three main peaks in the two TPR curves, but their intensities and positions are different. The two sets of peaks, P_2_, P_2_′ and P_3_, P_3_′, in the high-temperature range (> 300 °C) are attributed to the reduction of surface CeO_2_ and bulk CeO_2_, respectively [27], while the P_1_ and P_1_′ peaks in the low-temperature range (< 300 °C) are attributed to the reduction of PdO species and the Pd^2+^-O-Ce^4+^ structure [28]. The area of the P_1_ peak is significantly larger than that of the P_1_′ peak, which indicates that the calcined dealloyed sample contained more PdO species, which is consistent with the results of XPS. The fact that the P_1_ peak area is larger than that of the P_1_′ peak also indicates that for the calcined dealloyed sample, more of the Ce^4+^ ions connected with PdO species can be reduced at low temperatures, which is advantageous for the catalytic oxidation reaction. The P_2_ and P_2_′ peaks located near 470 °C also prove this conjecture. The area of the P_2_′ peak is significantly larger than that of the P_2_ peak, indicating that for the calcined dealloyed sample, the concentration of surface CeO_2_ species that can only be reduced at high temperatures was less than that of the dealloyed sample; in other words, a portion of the Ce^4+^ ions connected to PdO was reduced at low temperatures. Combined with the XPS analysis, the redox ability of the PdO/CeO_2_ composites is higher than that of the Pd/CeO_2_, which suggests that PdO/CeO_2_ composites exhibit a higher catalytic performance.

Based on the above characterization results, a possible formation mechanism for the PdO/CeO_2_ composites is proposed (Fig. 1). First, in the NaOH solution, Al is dissolved, and Ce reacts with OH^−^ at 80 °C to form the rod-like nanoporous Ce(OH)_3_ skeletal structure due to the anisotropy of Ce(OH)_3_ growth. At the same time, Pd atoms diffuse to the surface of the Ce(OH)_3_ nanorods. Because Ce(OH)_3_ is extremely unstable, it is easily dehydrated and oxidized to CeO_2_ during drying in air. After calcination under O_2_, most of the Pd nanoparticles on the surface of the CeO_2_ nanorods were oxidized to form PdO (Fig. 6b) and were partially embedded into the CeO_2_ nanorods (Fig. 4) at the high temperature to produce a strong metal-oxide-support interaction. As a result, the PdO/CeO_2_ composites were formed.

### Catalytic Activity Test

**Fig. 8:**
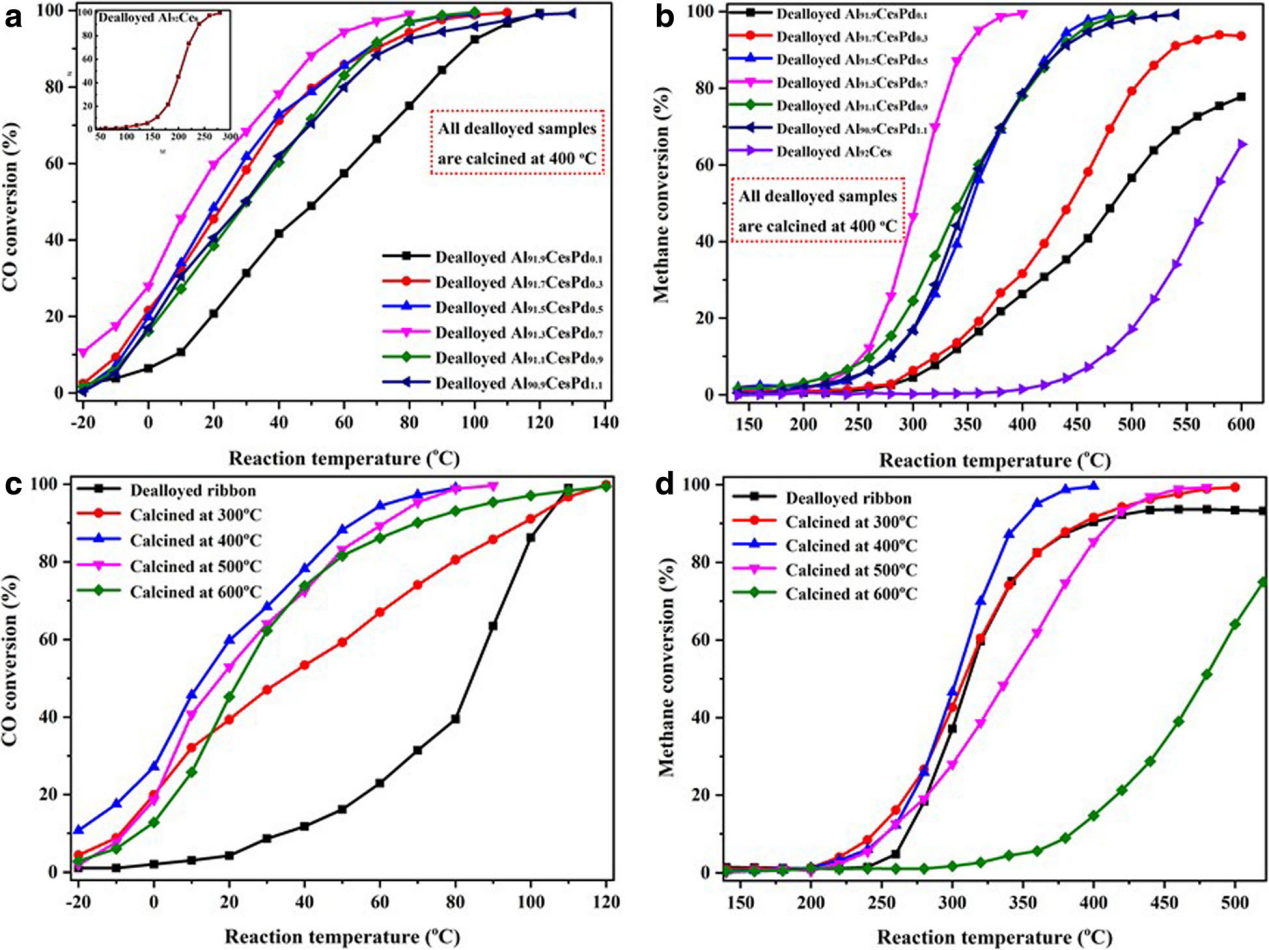
CO conversion (**a**) and CH_4_ conversion (**b**) as functions of the reaction temperature on the dealloyed Al_90_Ce_10_ ribbons and the dealloyed Al-Ce-Pd ribbons with different Pd contents calcined at 400 °C. The CO conversion (**c**) and CH_4_ conversion (**d**) as functions of the reaction temperature on the dealloyed Al_91.3_Ce_8_Pd_0.7_ calcined at different temperatures

The relationship between the content of Pd in the precursor and the catalytic activity of the catalysts is shown in Figs. 8 a and b. As shown in Fig. 8a, CeO_2_ nanorods exhibit a poor catalytic activity towards the oxidation of CO (inset), and the *T*_99_ (corresponding reaction temperature when the conversion is 99%) is as high as 280 °C. The catalytic activity was significantly increased for CO oxidation due to the loading of PdO. The sample of dealloyed Al_91.3_Ce_8_Pd_0.7_ showed the best CO catalytic activity, and the *T*_50_ (corresponding reaction temperature when the conversion is 50%) and *T*_99_ were 15 °C and 80 °C, respectively. The light-off temperature was also lower than − 20 °C. However, when the content of Pd in the precursor was further increased, the catalytic activity was slightly lowered. The catalytic activities towards CH_4_ combustion of the catalysts generated from precursors with different Pd contents are shown in Fig. 8b Similar to that seen for CO oxidation, the pure CeO_2_ nanorods exhibit a poor catalytic activity towards CH_4_ combustion, and the conversion at 600 °C was only 65%. After the addition of PdO, the catalytic activity was greatly improved. Similarly, the dealloyed Al_91.3_Ce_8_Pd_0.7_ sample exhibited the best catalytic activity towards CH_4_ combustion, with a light-off temperature of approximately 250 °C, and the *T*_50_ and *T*_99_ were 305 °C and 380 °C, respectively.

The CO and CH_4_ conversions as functions of the reaction temperature over dealloyed Al_91.3_Ce_8_Pd_0.7_ ribbons calcined at different temperatures are shown in Fig. 8c and d. The dealloyed ribbon (no calcination) exhibited a poor CO catalytic activity compared to that of the calcined samples, as shown in Fig. 8c. Combined with the XPS analysis, these results indicate that PdO supported on CeO_2_ nanorods exhibits a better CO catalytic activity than that of Pd, which is consistent with the H_2_-TPR analysis. Below 400 °C, the catalytic activity towards CO increased gradually with the calcination temperature; however, when calcined at temperatures greater than 400 °C, the catalytic activity towards CO decreased with increasing calcination temperature. For the CH_4_ combustion, similarly, the sample calcined at 400 °C exhibited the best catalytic activity, as shown in Fig. 8d. However, the dealloyed ribbon exhibited a light-off temperature and *T*_50_ similar to those of the calcined sample, and the conversion of CH_4_ was always lower than 93%. According to the experimental results and analysis of the catalytic activity of the dealloyed ribbon and the calcined dealloyed ribbon under different O_2_ atmospheres (Additional file 1: Figure S5), the reasons for this phenomenon may be because the light-off temperature for CH_4_ was high (> 240 °C) and a portion of the Pd had been oxidized into PdO; thus, the sample exhibits a good CH_4_ catalytic activity. However, because the composite is not calcined in a pure O_2_ atmosphere, the oxidation was insufficient to the extent that it is unable to fully convert CH_4_. For the dealloyed Al_91.3_Ce_8_Pd_0.7_ calcined at different temperatures, the order of catalytic activity towards CH_4_ is as follows: dealloyed sample (no calcination) < calcined at 600 °C < calcined at 500 °C < calcined at 300 °C < calcined at 400 °C. The experimental results show that the calcination temperature has an important influence on the catalytic activity of the sample.

**Fig. 9:**
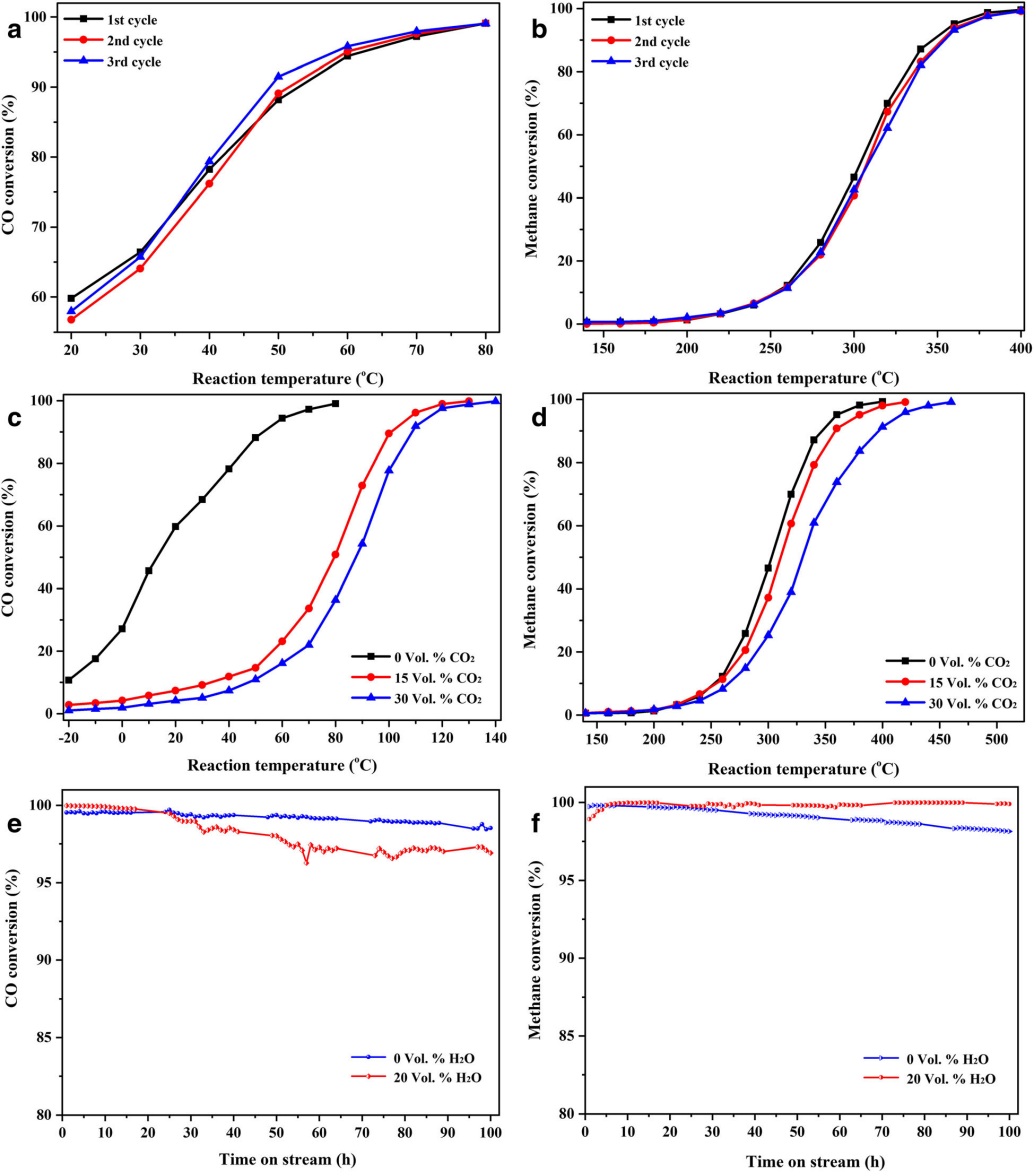
Cyclic stability tests (**a**, **b**), resistance to CO_2_ toxicity tests (**c**, **d**), and water resistance tests (**e**, **f**) of the dealloyed Al_91.3_Ce_8_Pd_0.7_ ribbons calcined at 400 °C

Cycle stability tests were also performed on the dealloyed Al_91.3_Ce_8_Pd_0.7_ ribbons calcined at 400 °C, as shown in Figs. 9a and b. Whether for the oxidation of CO or for the combustion of CH_4_, the results show that similar curves can be obtained for these three consecutive activity tests and that the catalyst is stable and not deactivated. This result indicates that the catalyst obtained by calcining dealloyed Al_91.3_Ce_8_Pd_0.7_ ribbons at 400 °C has practical application value and can be repeatedly used multiple times.

Generally, for catalysts with practical application value, they must be stable in the presence of CO_2_ and H_2_O. Reaction gases containing CO_2_ or H_2_O were passed over the catalyst to examine the CO_2_ and H_2_O tolerances of the dealloyed Al_91.3_Ce_8_Pd_0.7_ ribbons calcined at 400 °C, as shown in Figs. 9c–f. Compared with the response in the absence of CO_2_ in the reaction gas, the addition of 15 vol% CO_2_ reduced the activity of the catalyst towards CO oxidation, as shown in Fig. 9c, with a *T*_50_ and *T*_99_ of 80 °C and 130 °C, respectively. However, upon further increasing the CO_2_ to 30 vol%, the activity of the catalyst towards CO oxidation only slightly reduces, and the *T*_50_ and *T*_99_ are 88 °C and 140 °C, respectively. For methane combustion, the presence of 15 vol% CO_2_ in the reaction gas has little effect on the catalytic activity, and the *T*_50_ and *T*_99_ increased by only 5 °C and 30 °C, respectively, compared to those in the absence of CO_2_, as shown in Fig. 9d. When the concentration of CO_2_ was doubled (30 vol% CO_2_), the catalytic activity continues to decrease, with a *T*_50_ and *T*_99_ of 350 °C and 460 °C, respectively. Thus, in cases where the reactant concentration is constant, increasing the concentration of the CO_2_ products will form a strong competitive relationship with CO and CH_4_ for adsorption on the PdO nanoparticles and at its interfaces, thereby reducing the amount of CO or CH_4_ adsorbed per unit time and, consequently, the conversion rate. However, due to the higher reaction temperature required for methane combustion, the desorption of CO_2_ is enhanced such that the CO_2_ effect on methane combustion seems to be weaker than the effect on CO oxidation.

The long-term stability and water resistance tests of the catalyst are shown in Figs. 9e and f. For CO oxidation, the catalytic activity hardly decreases after 100 h of testing, regardless of the presence or absence of a high concentration of water vapour (20 vol%), indicating that the catalyst has excellent long-term stability and water resistance for CO oxidation. For methane combustion at high conversion (99%), the catalyst possesses similar properties with respect to CO oxidation. At the same time, the effect of water vapour on methane combustion at low conversion (30%, 50%, and 85%) is also discussed in Additional file 1: Figure S6. The effect of water vapour on the catalytic activity at low conversion is greater than that at high conversion. Detailed descriptions and discussions are presented in Additional file 1: Figure S6. The conclusions obtained are similar to those reported by Burch et al. [29]. By comparison with the water resistance of some recently reported Pd-based catalysts for methane combustion (Additional file 1: Table S2), the PdO/CeO_2_ catalyst prepared in this study retains a relatively excellent catalytic activity after a higher water vapour concentration (20 vol%) and a longer reaction time (100 h), which is very helpful to further the practical application of methane combustion.

**Fig. 10:**
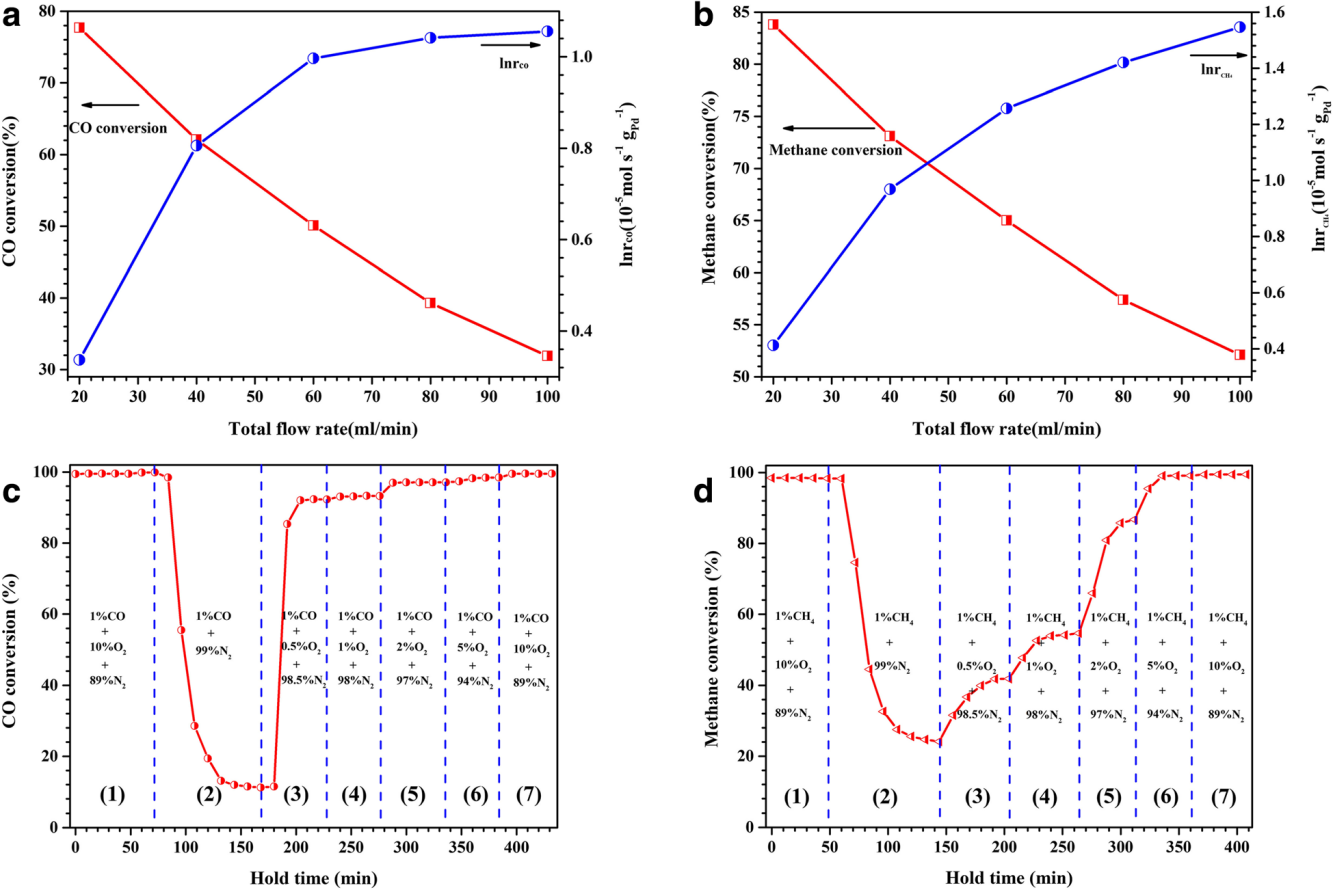
Catalytic activity and reaction rate of the dealloyed Al_91.3_Ce_8_Pd_0.7_ ribbons calcined at 400 °C for CO oxidation with different flow rates at 20 °C (**a**) and for methane combustion with different flow rates at 320 °C (**b**) (reaction gas 1 vol% CO/CH_4_, 10 vol% O_2_, and 89% vol% N_2_.). The catalytic activity of the dealloyed Al_91.3_Ce_8_Pd_0.7_ ribbons calcined at 400 °C for CO oxidation in the presence of different O_2_ concentrations at 80 °C (**c**) and for methane combustion in the presence of different O_2_ concentrations at 380 °C (**d**)

The flow rate of the reaction gas and the concentration of O_2_ are known to have an important influence on the catalytic activity of a catalyst. Here, the effect of the flow rate on the catalytic performance of the dealloyed Al_91.3_Ce_8_Pd_0.7_ ribbons calcined at 400 °C was first studied, as shown in Figs. 10a and b. As shown in Fig. 10a, for CO oxidation, when the total flow rate was increased from 20 mL min^−1^ (space velocity 12,000 h^−1^) to 100 mL min^−1^ (space velocity 60,000 h^−1^), the conversion of CO gradually decreased from the initial 78 to 31%. However, it is worth noting that although the conversion of CO decreased with increasing flow rate, the reaction rate (*r*_CO_) gradually increased. This phenomenon was caused by a decrease in the contact time of the gases with the active sites when the gas flow rate was increased, causing a decrease in the conversion; however, the actual amount of the reactant flowing over the active sites per unit time was increased, thereby resulting in the increased reaction rate. A similar phenomenon was observed for methane combustion. However, it appears that the flow rate had a slightly smaller effect on the conversion of CH_4_ than on the conversion of CO. As the flow rate increased from 20 to 100 mL min^−1^, the conversion of methane decreased from an initial value of 84 to 53%. Moreover, the reaction rate of methane combustion (*r*_CH4_) exhibited a linear increase with the flow rate, rather than tending to remain stable, similar to that seen for *r*_CO_. This indicates that the catalyst has a wider flow rate range for methane combustion than for CO oxidation. In this study, the calculated values of *r*_CO_ and *r*_CH4_ under different test conditions were in the range of 1.40~2.87 $\left(\times {10}^{-5}\mathrm{mol}\cdotp {\mathrm{g}}_{\mathrm{Pd}}^{-1}\cdotp {\mathrm{s}}^{-1}\right)$and 1.51~4.70 $\left(\times {10}^{-5}\mathrm{mol}\cdotp {\mathrm{g}}_{\mathrm{Pd}}^{-1}\cdotp {\mathrm{s}}^{-1}\right)$, respectively.

Next, the effect of the O_2_ concentration in the reaction gas on the catalytic activity of the catalyst was investigated. For CO oxidation, as shown in Fig. 10c, when under oxygen-rich conditions (10 vol% O_2_), the CO conversion was maintained at 99%, and as the O_2_ concentration suddenly decreased to 0 (anaerobic conditions), the CO conversion decreased rapidly before eventually stabilizing at approximately 12%. The reason for this phenomenon was that the surface lattice oxygen participated in the oxidation reaction of CO. Generally, the CO oxidation pathway involving lattice oxygen on the surface of the support is slow and inefficient compared to the direct adsorption activation of the O_2_ molecule [30]. Therefore, the CO conversion remained at a lower level under the anaerobic conditions in this study. This result also indicated that the CeO_2_ carrier has a strong ability to store/release oxygen. Subsequently, 0.5% O_2_ was introduced into the reaction gas, and the CO conversion rapidly recovered to 90%. As the O_2_ concentration continues to increase, the CO conversion eventually reached the initial 99%, and a new steady state was established. For methane combustion (Fig. 10d), a similar result to that of CO oxidation was observed but with two different points. The first point was that when in an anaerobic environment, the conversion of CH_4_ finally stabilized at 25%, higher than that of the anaerobic conversion of CO, which indicated that a high reaction temperature could accelerate the migration of surface lattice oxygen thereby improving the conversion efficiency. The second point was that as the O_2_ concentration increased, the increasing rate of CH_4_ conversion and the final establishment of a steady state were slower than those in the CO conversion, which may be due to the incomplete combustion of methane under oxygen-poor conditions (0.5~2 vol% O_2_). This result also shows that methane combustion is a more complicated and difficult reaction compared to CO oxidation.

**Fig. 11:**
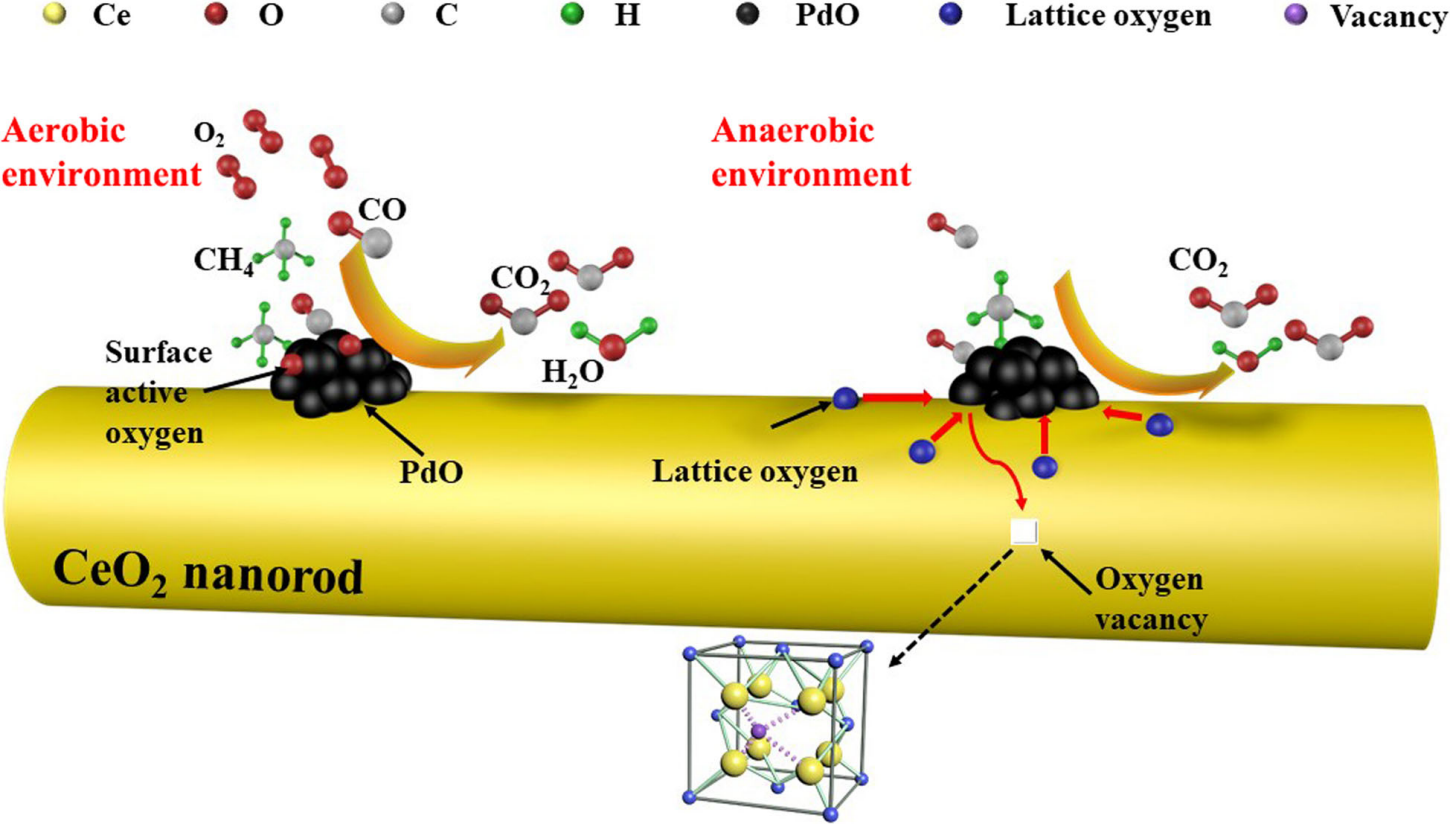
Schematic illustration of CO oxidation and methane combustion over the rod-like nanoporous PdO/CeO_2_ catalysts

Based on the results of the characterization and experiments, a simple mechanism for CO oxidation and methane combustion is proposed, as shown in Fig. 11.

First, the CO and CH_4_ molecules in the reaction gas are adsorbed onto the surface of PdO, reacting rapidly with the adsorbed and activated oxygen on the surface of PdO, and then CO_2_ and H_2_O are produced and desorbed. The active sites become available again, and a high reaction rate for CO oxidation and CH_4_ combustion is maintained. It is worth noting that the catalytic oxidation reaction can still proceed slowly under anaerobic conditions, which is shown to be related to the participation of the surface lattice oxygen of the nanorods in the catalytic reaction (Figs. 10c, d), as shown in Fig. 11.

A large number of experimental results indicated that the PdO/CeO_2_ catalyst prepared by dealloying combined with calcination exhibited excellent catalytic activities towards CO oxidation and methane combustion and possesses outstanding cycle stability, resistance to CO_2_ toxicity, and water resistance. In addition to its inherent simplicity, the “green” preparation method of dealloying can effectively avoid the contamination of nanomaterials by organic chemicals and other surfactants, which are common to wet chemical synthesis methods. In addition, the PdO/CeO_2_ catalyst prepared by dealloying combined with calcination exhibits excellent reproducibility, and the repeated experiments detailed in Additional file 1: Figures S7–S10, Tables S3 and S4 proved this point very well. Therefore, this work can provide insight into the preparation of other new catalysts.

## Conclusions

In summary, a simple method of dealloying an Al-Ce-Pd ribbon combined with calcination has been developed for the preparation of a PdO/CeO_2_ rod-like nanoporous composite. The experimental results indicate that the sample prepared by the dealloying of an Al_91.3_Ce_8_Pd_0.7_ ribbon in 20 wt% solution and then calcining at 400 °C showed the best catalytic activities towards CO oxidation and methane combustion, and the reaction temperatures for the complete conversions of CO and CH_4_ are 80 °C and 380 °C, respectively. The high catalytic activities could be attributed to the good dispersion of the PdO nanoparticles (having a large specific surface area of 102 m^2^ g^−1^), a strong redox capacity, the interaction between PdO and CeO_2_, and more surface active oxygen on PdO. In addition, the catalyst also exhibited excellent cycle stability, resistance to CO_2_ toxicity, and water resistance, where after 100 h of testing, the catalytic activity hardly decreased in the presence of H_2_O. Furthermore, the catalytic reactions can occur even under anaerobic conditions. These results demonstrate the feasibility of the combined dealloying calcination method for the preparation of new catalysts. It is expected that the method can be applied to the preparation of similar composite materials.

## Additional file

Additional file 1: **Figure S1**. Apparatus related to the addition of H_2_O. **Figure S2**. SEM (a, c, e, g, i) and TEM (b, d, f, h, j) images of the dealloyed Al_91.3_Ce_8_Pd_0.7_ under different calcination temperatures. **Figure S3**. XPS spectrum of Ce 3d region of dealloyed Al_92_Ce_8_ sample calcined at 400 °C. **Figure S4**. XPS spectra of the Pd 3d (a) and O 1s (b) region of the dealloyed Al_91.3_Ce_8_Pd_0.7_ samples were calcined at 400 °C in vacuum (Pd/CeO_2_) and O_2_ atmosphere (PdO/CeO_2_), respectively. **Figure S5**. Catalytic activity of calcined samples (a) and uncalcined samples (b) at different O_2_ contents for methane combustion. **Figure S6**. Effect of water vapour at different temperatures on the activity for methane combustion over dealloyed Al_91.3_Ce_8_Pd_0.7_ calcined at 400 °C. **Figure S7**. Pore size distribution curves of dealloyed Al_91.3_Ce_8_Pd_0.7_ calcined at different temperatures in the repeated experiment. **Figure S8**. XPS spectra of the Ce 3d (a), Pd 3d (b), and O 1s (c) region of dealloyed Al_91.3_Ce_8_Pd_0.7_ sample and dealloyed Al_91.3_Ce_8_Pd_0.7_ sample calcined at 400 °C in the repeated experiment. **Figure S9**. XPS spectrum of Ce 3d region of dealloyed Al_92_Ce_8_ sample calcined at 400 °C in the repeated experiment (b). **Figure S10**. CO conversion (a) and CH_4_ conversion (b) as functions of the reaction temperature on the dealloyed Al_91.3_Ce_8_Pd_0.7_ ribbons calcined at 400 °C. **Table S1**. Crystalline size calculated by the Scherrer equation for the dealloyed Al_91.3_Ce_8_Pd_0.7_ calcined at different temperatures. **Table S2**. Water resistance data of several Pd-based catalysts for methane combustion. **Table S3**. Specific surface area (*S*_BET_), pore size (*D*_p_), and pore volume (*V*_p_) of dealloyed Al_91.3_Ce_8_Pd_0.7_ ribbons calcined at different temperatures and the average and variance. **Table S4**. Ratios of Ce, Pd, and O in different states for different catalysts as obtained from XPS results and the average and variance. (DOCX 11759 kb)

## Data Availability

The datasets supporting the conclusions of this article are included within the article.

## Abbreviations

CH_4_: Methane
CO: Carbon monoxide
*D*_p_: Pore size
EDS: Energy dispersive spectrometer
EDX: Energy-dispersive X-ray spectra
FID: Flame ionization detector
H_2_-TPR: Hydrogen temperature-programmed reduction
*S*_BET_: Specific surface areas
SEM: Scanning electron microscope
TEM: Transmission electron microscope
*V*_p_: Pore volume
XPS: X-ray photoelectron spectroscopy
XRD: X-ray diffraction
