# Supplementary material for: Rod-Like Nanoporous CeO2 Modified by PdO Nanoparticles for CO Oxidation and Methane Combustion with High Catalytic Activity and Water Resistance
Source: Nanoscale Res Lett. 2019 Jun 6;14:199. doi: 10.1186/s11671-019-3029-4 (PMC6554377; doi:10.1186/s11671-019-3029-4)
Supplement: Supplementary file 1 — Figure S1. Apparatus related to the addition of H2O. Figure S2. SEM (a, c, e, g, i) and TEM (b, d, f, h, j) images of the dealloyed Al91.3Ce8Pd0.7 under different calcination temperatures. Figure S3. XPS spectrum of Ce 3d region of dealloyed Al92Ce8 sample calcined at 400 °C. Figure S4. XPS spectra of the Pd 3d (a) and O 1s (b) region of the dealloyed Al91.3Ce8Pd0.7 samples were calcined at 400 °C in vacuum (Pd/CeO2) and O2 atmosphere (PdO/CeO2), respectively. Figure S5. Catalytic activity of calcined samples (a) and uncalcined samples (b) at different O2 contents for methane combustion. Figure S6. Effect of water vapour at different temperatures on the activity for methane combustion over dealloyed Al91.3Ce8Pd0.7 calcined at 400 °C. Figure S7. Pore size distribution curves of dealloyed Al91.3Ce8Pd0.7 calcined at different temperatures in the repeated experiment. Figure S8. XPS spectra of the Ce 3d (a), Pd 3d (b), and O 1s (c) region of dealloyed Al91.3Ce8Pd0.7 sample and dealloyed Al91.3Ce8Pd0.7 sample calcined at 400 °C in the repeated experiment. Figure S9. XPS spectrum of Ce 3d region of dealloyed Al92Ce8 sample calcined at 400 °C in the repeated experiment (b). Figure S10. CO conversion (a) and CH4 conversion (b) as functions of the reaction temperature on the dealloyed Al91.3Ce8Pd0.7 ribbons calcined at 400 °C. Table S1. Crystalline size calculated by the Scherrer equation for the dealloyed Al91.3Ce8Pd0.7 calcined at different temperatures. Table S2. Water resistance data of several Pd-based catalysts for methane combustion. Table S3. Specific surface area (SBET), pore size (Dp), and pore volume (Vp) of dealloyed Al91.3Ce8Pd0.7 ribbons calcined at different temperatures and the average and variance. Table S4. Ratios of Ce, Pd, and O in different states for different catalysts as obtained from XPS results and the average and variance. (DOCX 11759 kb) [file 11671_2019_3029_MOESM1_ESM.docx]

**Rod-like nanoporous CeO_2_ modified by PdO nanoparticles for CO oxidation and methane combustion with high catalytic activity and water resistance**

Dong Duan^a, b, c^, Chunxi Hao^a, b, c^, Liqun Wang^a, b, c^, Wenyu Shi^a, b, c^, Haiyang Wang^a, b, c^, Gege He^a, b, c^, Lumei Gao^a, b, c^, and Zhanbo Sun^a, b, c,*^

^a^ *School of Science, MOE Key Laboratory for Non-Equilibrium Synthesis and Modulation of Condensed Matter,* *Xi'an Jiaotong University, Xi'an, 710049, PR China.*

^b^ *State Key Laboratory for Mechanical Behavior of Materials, Xi'an Jiaotong University, Xi'an, 710049, PR China.*

^c^ *Key Laboratory of Shaanxi for Advanced Functional Materials and Mesoscopic Physics, Xi'an Jiaotong University, Xi'an, 710049, PR China.*

^*^ *Corresponding author: Zhanbo Sun. Email:* *[szb@mail.xjtu.edu.cn](mailto:szb@mail.xjtu.edu.cn).*

*Email addresses for all authors:* *Dong Duan (*[*15529287317@163.com*](mailto:15529287317@163.com)*); Chunxi Hao (*[*1454232576@qq.com*](mailto:1454232576@qq.com)*); Liqun Wang (*[*wanglq@xjtu.edu.cn*](mailto:wanglq@xjtu.edu.cn)*); Wenyu Shi (*[*924746566@qq.com*](mailto:924746566@qq.com)*); Haiyang Wang (*[*1264962862@qq.com*](mailto:1264962862@qq.com)*); Gege He (*[*gghe01@163.com*](mailto:gghe01@163.com)*); Lumei Gao (*[*lmgao@mail.xjtu.edu.cn*](mailto:lmgao@mail.xjtu.edu.cn)*).*


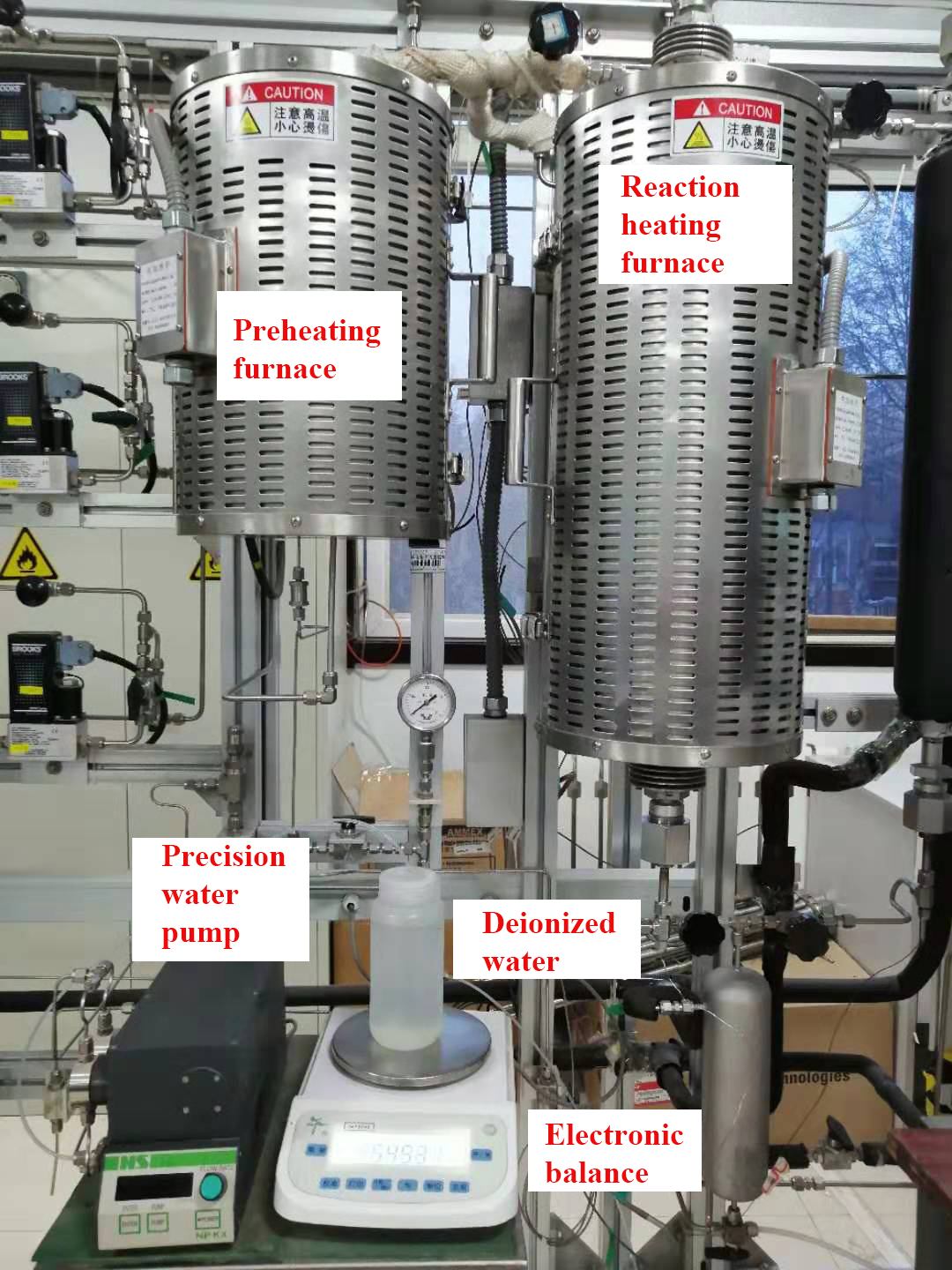


Fig. S1 Apparatus related to the addition of H_2_O.

Fig. S1 is an apparatus related to the addition of H_2_O, which consists of deionized water, an electronic balance, a precision water pump, a preheating furnace and a reaction heating furnace. First, deionized water is added to the sealed bottle, and then the deionized water is transferred to the preheating furnace (preheating temperature: 383 K) through a precision water pump that can control the flow rate, and mixed with the reaction gas (CO+O_2_+N_2_). The flow rate of deionized water is 7.5μL/min. Electronic balances are used to observe whether the precision water pump is working properly through the change in the quality of the deionized water. After the water vapour and the reaction gas are thoroughly mixed in the preheating furnace, they are feed into the reaction heating furnace together. Finally, the H_2_O is recovered through the drying unit (because H_2_O cannot enter the gas chromatograph), and the conversion is recorded by gas chromatography.


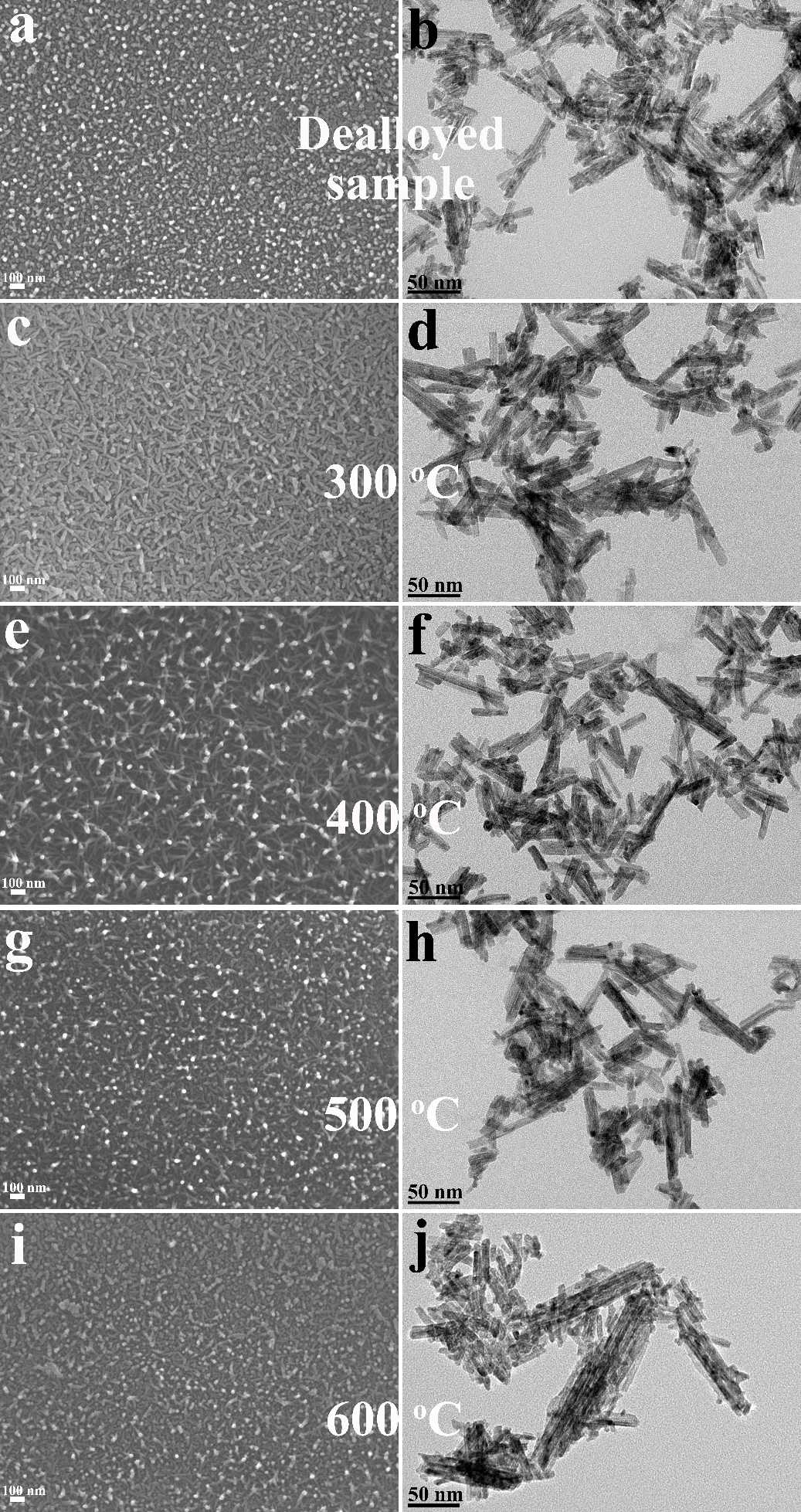


Fig. S2 SEM (a, c, e, g, i) and TEM (b, d, f, h, j) images of the dealloyed Al_91.3_Ce_8_Pd_0.7_ under different calcination temperatures.

Table S1 crystalline size calculated by the Scherrer equation for the dealloyed Al_91.3_Ce_8_Pd_0.7_ calcined at different temperatures.

| **Calcination temperature (°C)** | **Crystalline size (nm)** |
| --- | --- |
| Calcined at 200 °C | 6.2 nm |
| Calcined at 400 °C | 5.9 nm |
| Calcined at 600 °C | 6.8 nm |
| Calcined at 800 °C | 7.9 nm |

Table S1 shows the crystalline size of the samples calcined at different temperatures calculated by the Scherrer equation. It can be seen that as the calcination temperature increases, the crystalline size increases only slightly (from about 6 nm to about 8 nm), which indicates that the CeO_2_ skeletal structure can stabilize the crystalline size well. The morphology of the samples calcined at different temperatures obtained by direct observation by SEM and TEM is shown in Fig. S2. Similar to the results calculated by the Scherrer equation, as the calcination temperature increases, no significant increase in the diameter of the nanorods is observed.

The results of the above experiments and analyses support the view of "skeletal structure has an excellent anti-sintering ability" in the article.

*
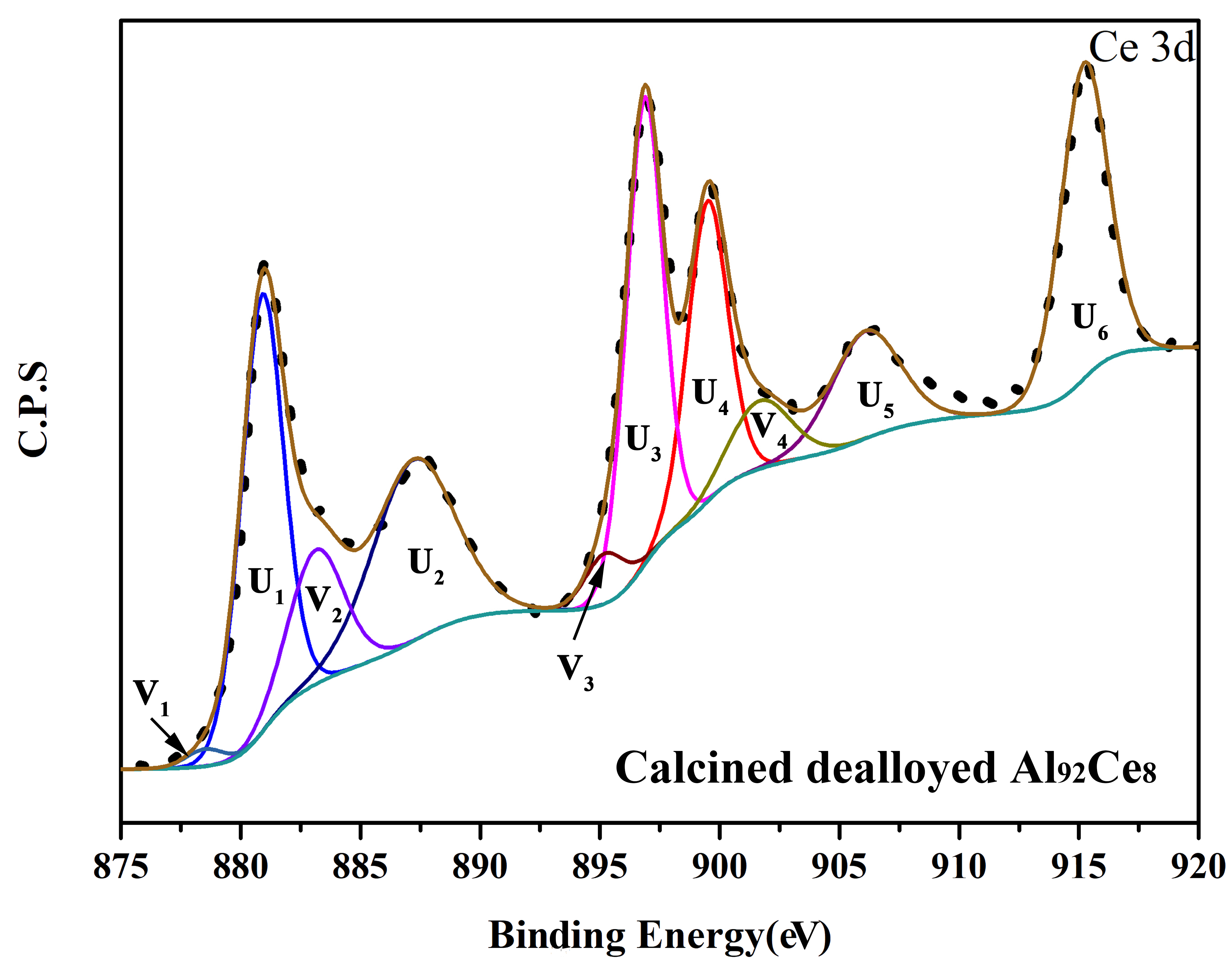
*

Fig. S3 XPS spectrum of the Ce 3d region of the dealloyed Al_92_Ce_8_ sample calcined at 400 °C.


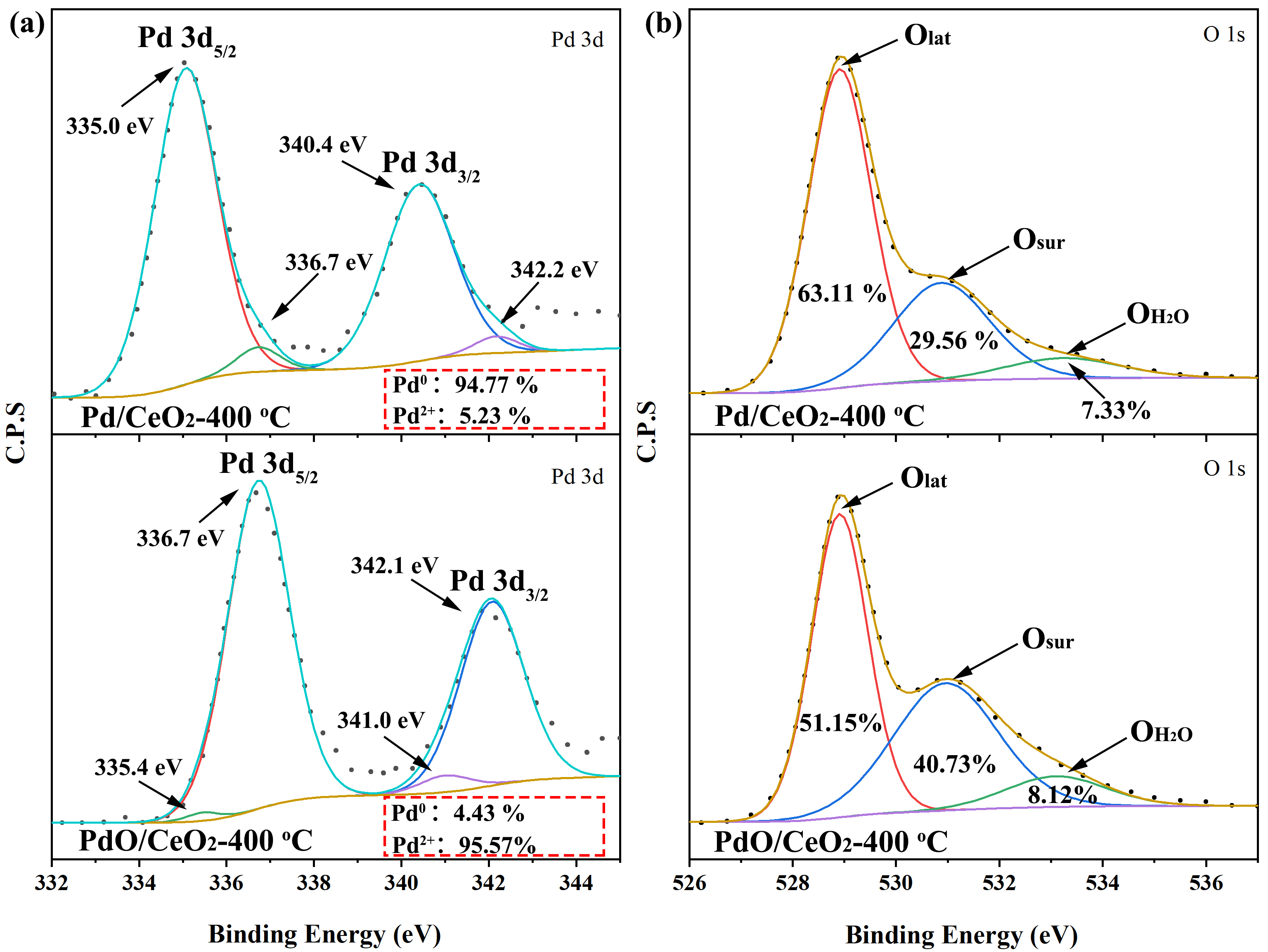


Fig. S4 XPS spectra of the Pd 3d (a) and O 1s (b) region of the dealloyed Al_91.3_Ce_8_Pd_0.7_ samples were calcined at 400 ^o^C in vacuum (Pd/CeO_2_) and O_2_ atmosphere (PdO/CeO_2_), respectively.

It is known that thermal activation is important for oxygen chemisorption. Therefore, In order to better support the view of “PdO has a stronger ability to adsorb and activate O_2_ than do the metallic Pd nanoparticles” in the article, the dealloyed Al_91.3_Ce_8_Pd_0.7_ samples were calcined at 400 ^o^C in vacuum and O_2_ atmosphere respectively to eliminate the effect of thermal activation for oxygen chemisorption, at the same time, it is ensured that the Pd elements in the two samples exist in the form of metals Pd (Pd^0^) and PdO (Pd^2+^), respectively. The calcined two samples were pretreated in a 10 % O_2_ (O_2_: N_2_ = 1: 9) atmosphere at room temperature for 1 h to simulate the oxygen chemisorption during the reaction, and ensuring the adsorption procedure was conduct in the same condition, the result was shown in Fig. S4. It can be seen that Pd/CeO_2_ and PdO/CeO_2_ composites can be obtained by calcination in a vacuum and O_2_ atmosphere, respectively. For Pd/CeO_2_ samples (calcined in the vacuum), the content of surface active oxygen species (29.56 %) is still lower than that of PdO/CeO_2_ (40.73 %), as shown in Fig. S4 (b), which is consistent with the conclusions in the article, that is, PdO has a stronger ability to adsorb and activate O_2_ than do the metallic Pd nanoparticles.


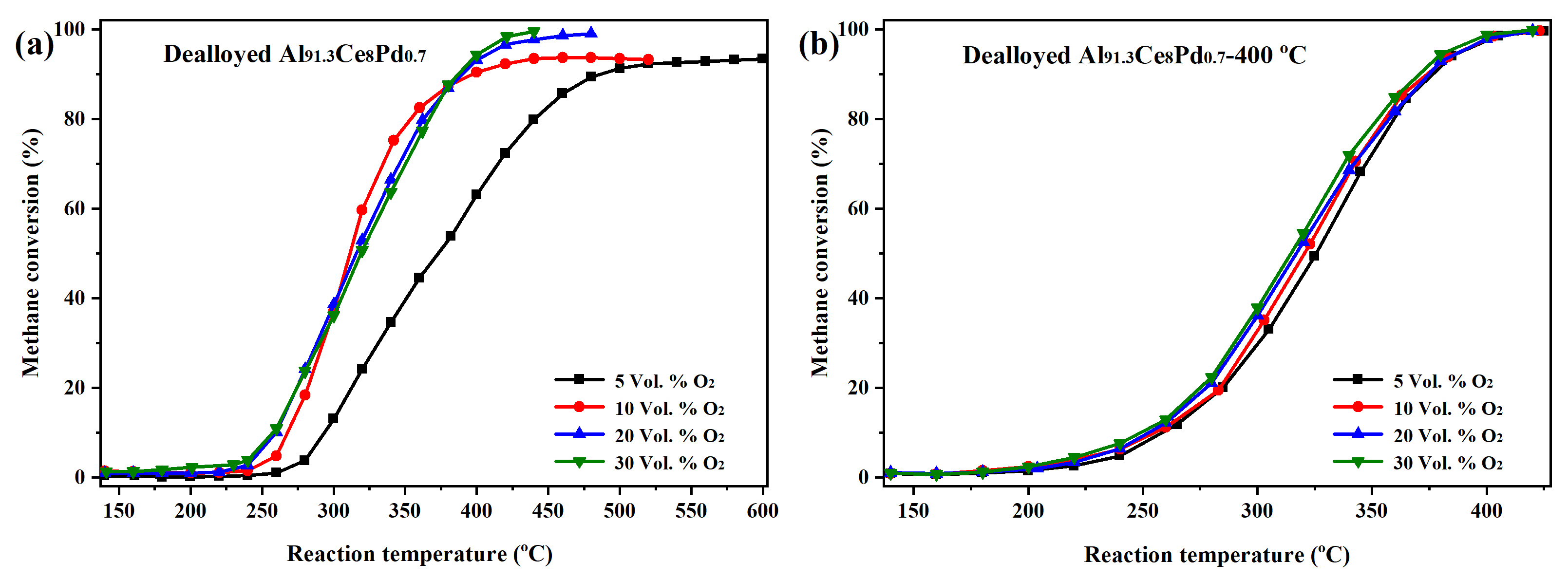


Fig. S5 Catalytic activity of calcined samples (a) and uncalcined samples (b) at different O_2_ contents for methane combustion.

In order to explore the reason why the methane conversion of the uncalcined sample is always lower than 93%, the following experiment was designed.

The calcined sample and the uncalcined sample were respectively placed in a reaction gas of 5% O_2_, 10% O_2_, 20% O_2_ and 30% O_2_, in order to test the catalytic activity of two samples for methane at different O_2_ contents，as shown in Fig. S5. It can be seen that several very similar activity curves were obtained in the reaction gases with different O_2_ contents for the calcined sample (PdO/CeO_2_), which indicates that for the calcined sample (PdO/CeO_2_), the O_2_ content in reaction gas does not affect the catalytic activity of the sample under oxygen-rich conditions, Fig. 10 (d) also proves this viewpoint (when the O_2_ content increases from 5% to 10%, the catalytic activity hardly changes).

However, for the uncalcined sample (Pd/CeO_2_), it can be seen that the content of O_2_ in the reaction gas has a great influence on the catalytic activity of the sample, the catalytic activity increases as the O_2_ content increases, and, the phenomenon that CH_4_ could not be completely converted was observed in the case of 5% and 10% O_2_. Combined with the results of XPS, we believe that this is related to the insufficient oxidation of the uncalcined sample during the relatively low O_2_ content (5% and 10%) test. And the O_2_ flow rate (15 mL/min) at 30% was close to the O_2_ flow rate (18 mL/min) at the time of calcination, therefore, the catalytic activity of the uncalcined sample at the 30% O_2_ content is close to that of the calcined sample.

Based on this, we put forward the viewpoint in the article, namely “ the light-off temperature of CH_4_ was high (＞240 °C), and a portion of the Pd has been oxidized into PdO, thus it exhibits good CH_4_ catalytic activity. However, because it is not calcined in a pure O_2_ atmosphere, the oxidation was insufficient, such that it is unable to fully convert CH_4_ ”.


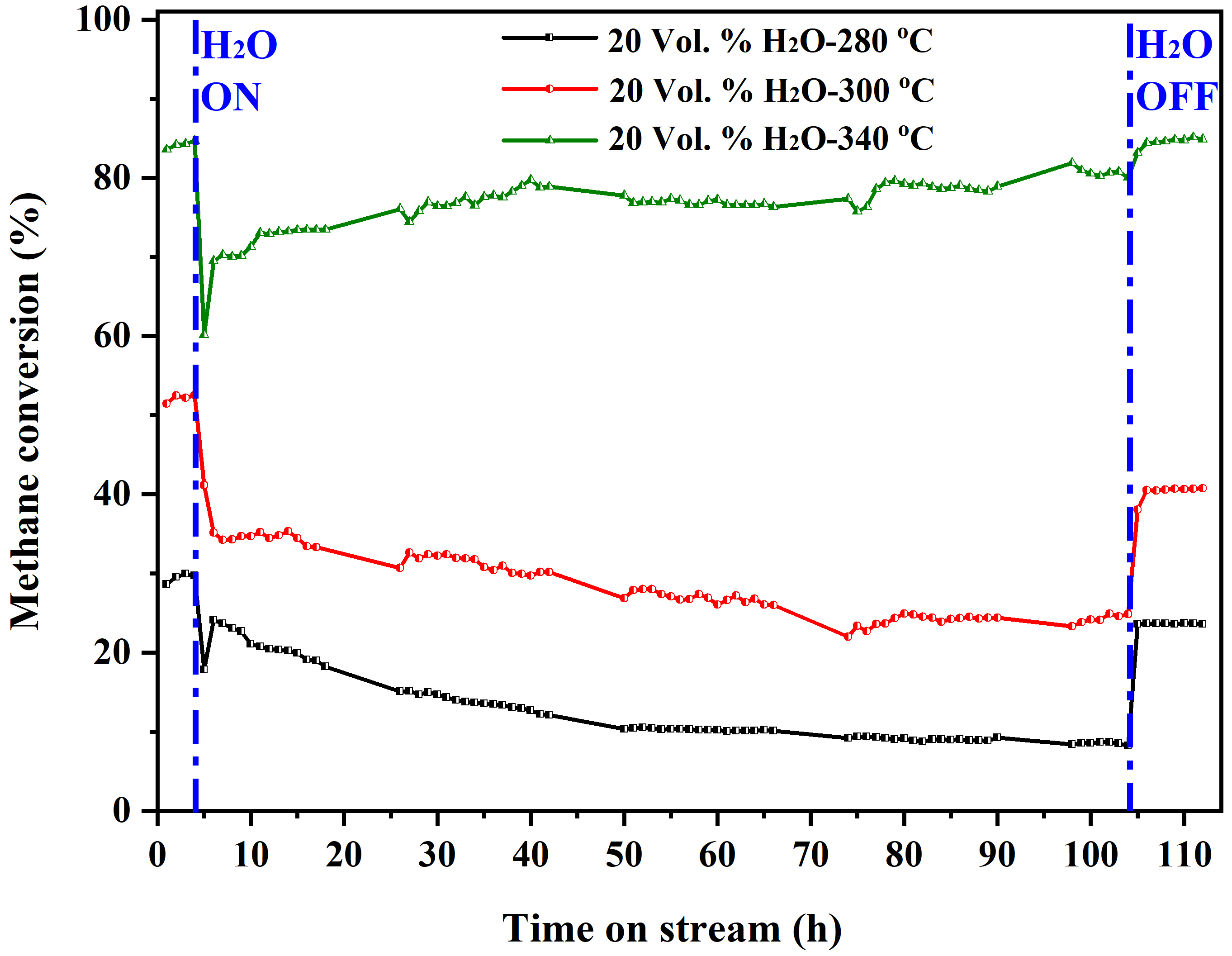


Fig. S6 Effect of water vapour at different temperatures on the activity for methane combustion over dealloyed Al_91.3_Ce_8_Pd_0.7_ calcined at 400 °C.

Table S2 Water resistance data of several Pd-based catalysts for methane combustion.

| **Sample** | **Water content (%)** | **time on stream**  **(h)** | **Conversion temperature**  **(^o^C)** | | **Conversion rate**  **(%)** | | | **Reference** |
| --- | --- | --- | --- | --- | --- | --- | --- | --- |
|  |  |  | T_50_ | T_90_ | Before adding water | During adding water | After removing water |  |
| Pd/Al_2_O_3_ | 2.7 | 0.25 | 330 | 400 | 90 | 72 | 91 | [[1](#_ENREF_7)] |
|  |  |  |  |  | 60 | 25 | 53 |  |
|  |  |  |  |  | 20 | 3 | 18 |  |
| Au-Pd/3DOM Co_3_O_4_ | 5 | 22 | 337 | 379 | 52 | 47 | 52 | [[2](#_ENREF_8)] |
| Pd@CeO_2_/Si-Al_2_O_3_ | 15 | 4 | / | / | 90 | 60 | 98 | [[3](#_ENREF_9)] |
| PdO/CeO_2_ on SBA-15 | 2 | 19 | 290 | 330 | 85 | 50 | 95 | [[4](#_ENREF_10)] |
|  | 5 |  |  |  | 85 | 18 | 95 |  |
| PdO/CeO_2_ | 20 | 100 | 300 | 345 | 30 | 10 | 25 | This work |
|  |  |  |  |  | 50 | 25 | 40 |  |
|  |  |  |  |  | 85 | 80 | 85 |  |

The effect of water vapour on different methane conversion levels may be different, as shown in Fig. S6. It can be seen that at lower conversion (30%), the methane conversion gradually decreases with the addition of water. After about 50 h, the methane conversion tended to be stable, and it was about 10% at this time. After 100 h, the water was removed and the conversion quickly recovered to around 25%, and the conversion lost only 5% compared to the initial conversion. The 50% conversion of methane (300 ^o^C) is similar to the conversion of 30%, but it is noted that after the water is removed, the methane conversion is reduced to about 40 %, and the conversion is lost by 10%, which is higher than the former case (30 % conversion). When the conversion reached 85% (340 ^o^C), the conversion began to decrease with the addition of water, and it stabilized after about 40 h. At this time, the conversion is only slightly lower than the initial conversion (85%). After the water was removed, the conversion returned to about 85%, with almost no decrease. This indicates that the effect of water on methane conversion is little or even negligible at high reaction temperatures (high conversion).

Compared with the known literature, the PdO/CeO_2_ catalyst prepared by combining dealloying with calcination method retains a relatively excellent catalytic activity after a higher H_2_O concentration (20%) and longer reaction time, as shown in Table S2, this is very helpful for further practical application of methane combustion.


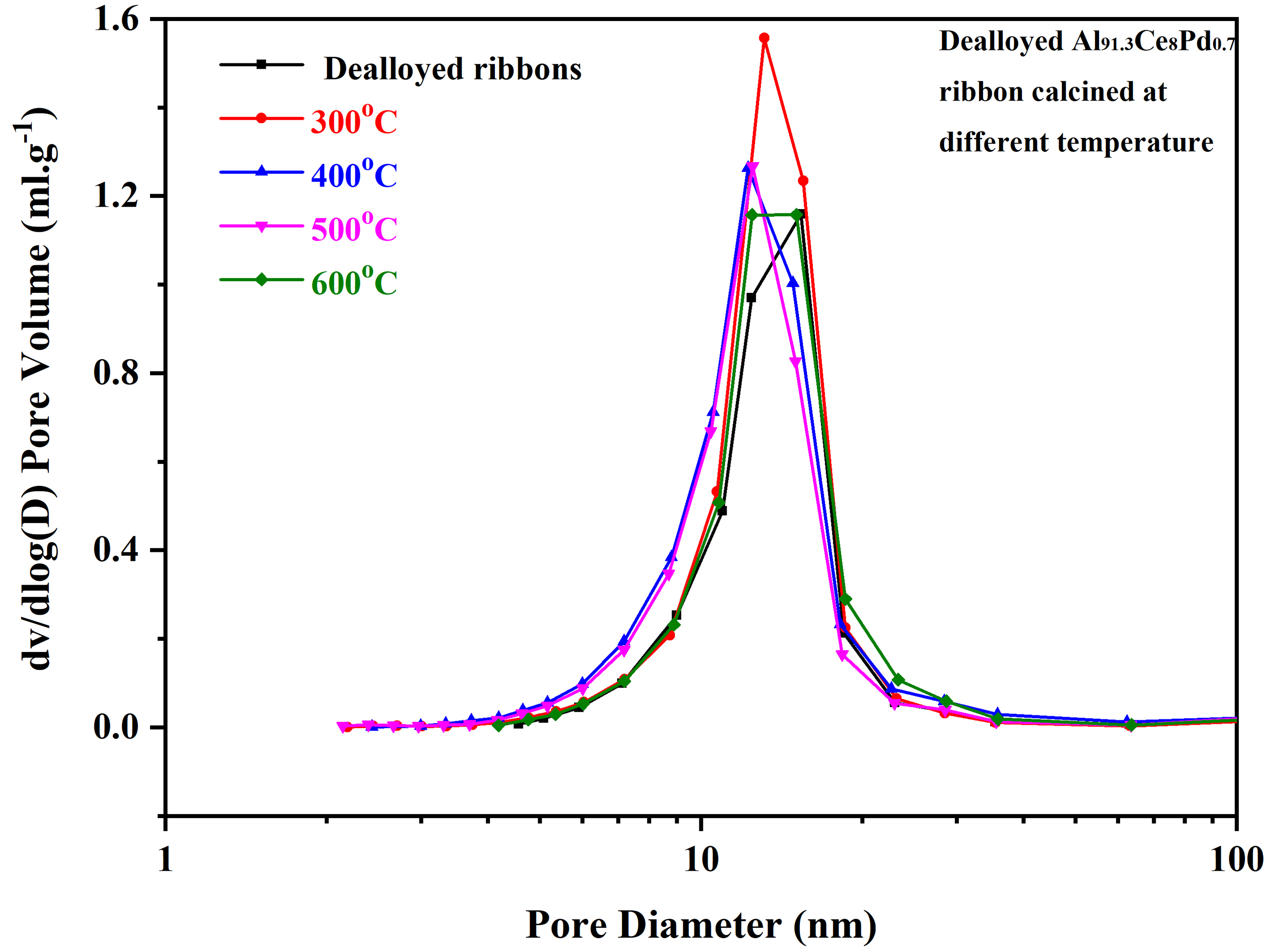


Fig. S7 Pore-size distribution curves of dealloyed Al_91.3_Ce_8_Pd_0.7_ ribbons calcined at different temperatures in the repeated experiment.


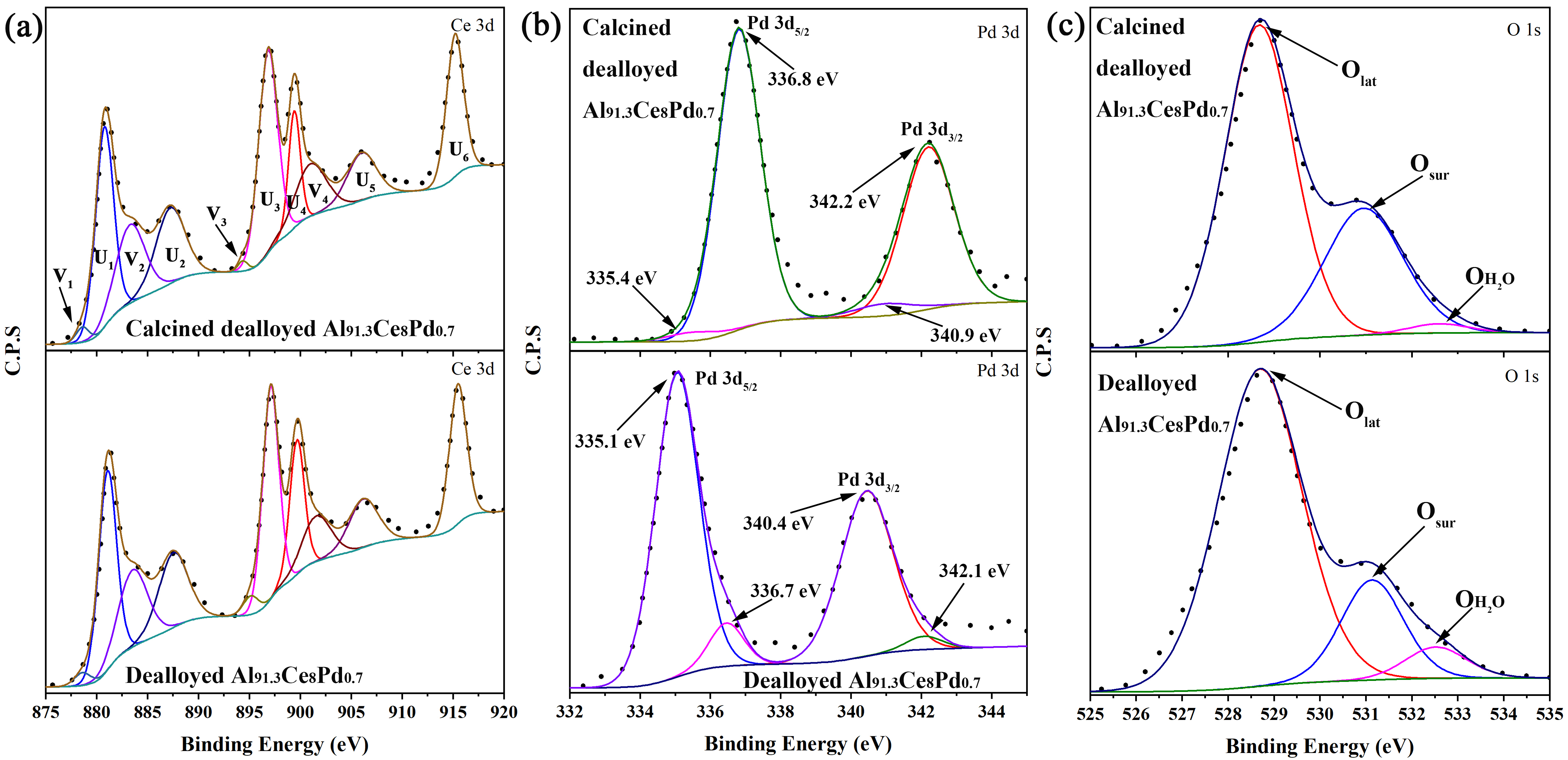


Fig. S8 XPS spectra of the Ce 3d (a), Pd 3d (b) and O 1s (c) region of the dealloyed Al_91.3_Ce_8_Pd_0.7_ sample and the dealloyed Al_91.3_Ce_8_Pd_0.7_ sample calcined at 400 °C in the repeated experiment.


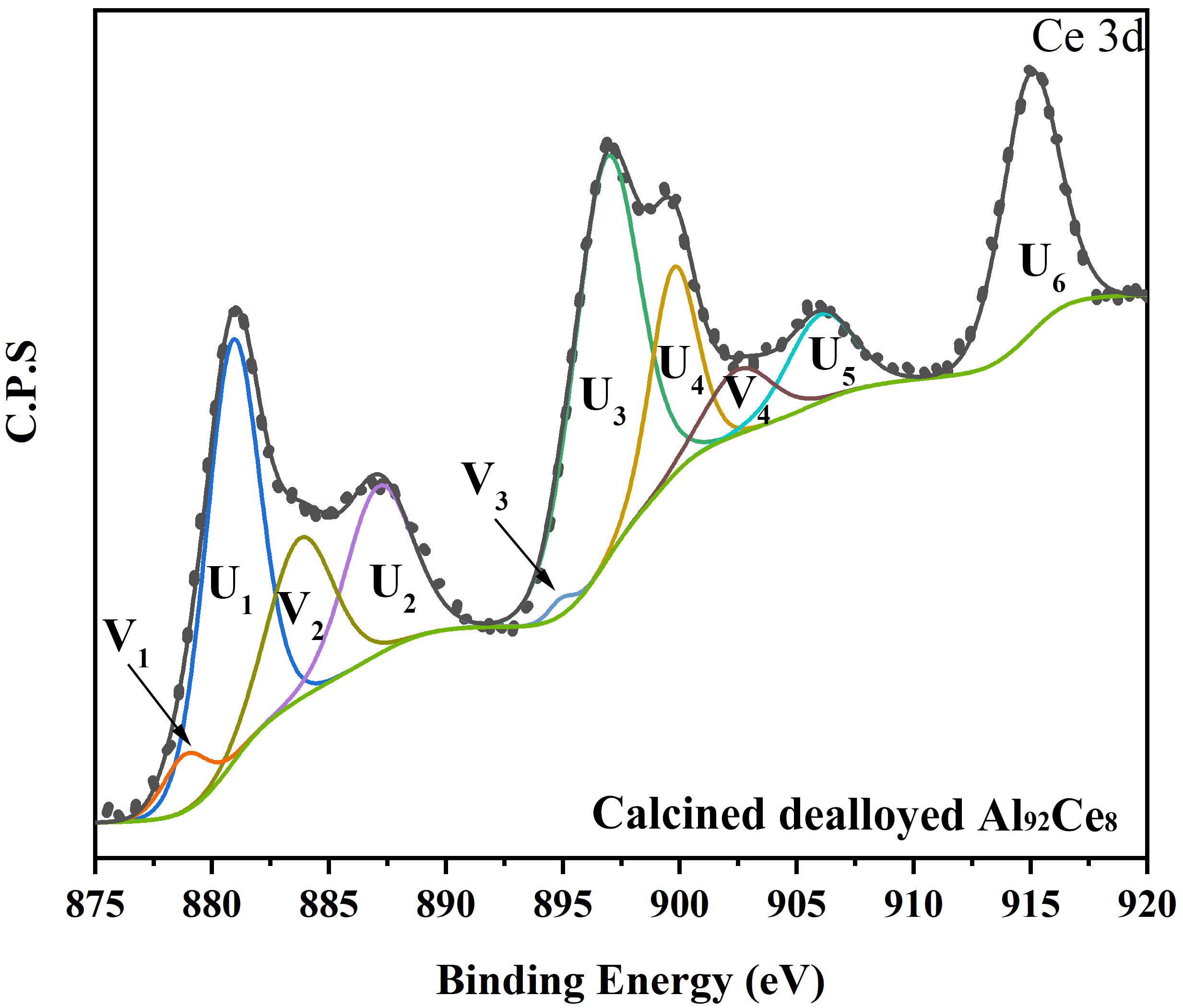


Fig. S9 XPS spectrum of the Ce 3d region of the dealloyed Al_92_Ce_8_ sample calcined at 400 °C in the repeated experiment (b).


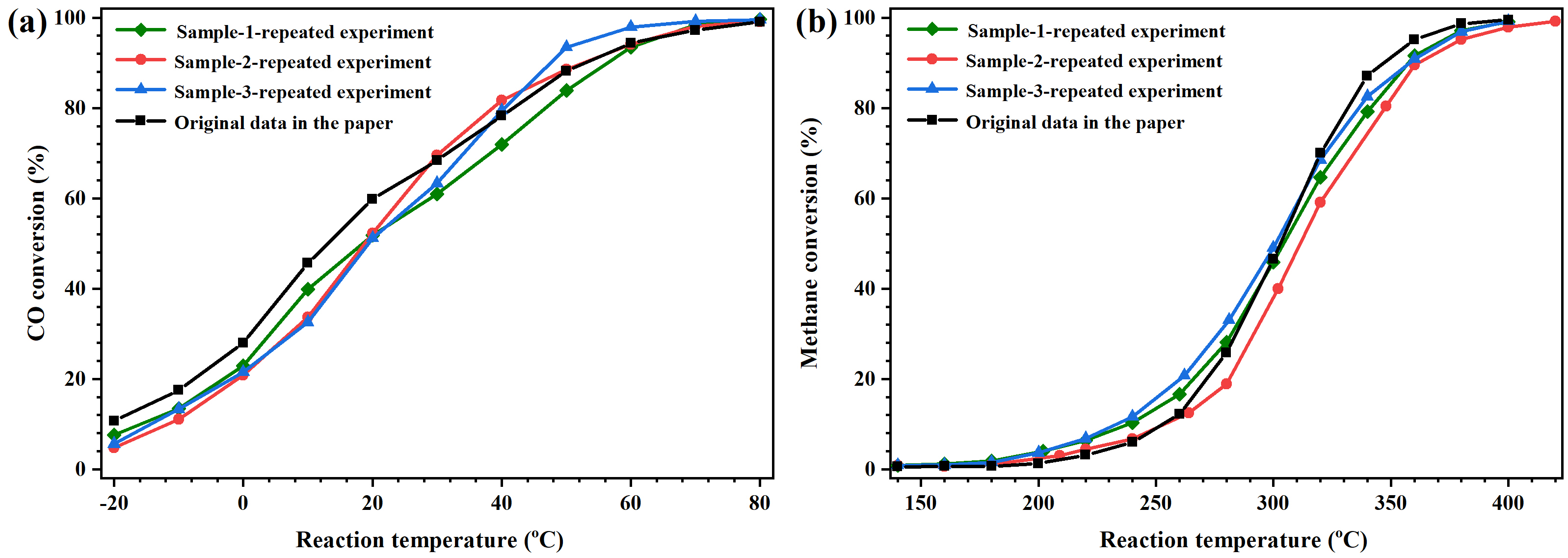


Fig. S10 CO conversion (a) and CH_4_ conversion (b) as functions of the reaction temperature on the dealloyed Al_91.3_Ce_8_Pd_0.7_ ribbons calcined at 400 °C.

Table S3 Specific surface area (S_BET_), pore size (D_p_) and pore volume (V_p_) of the dealloyed Al_91.3_Ce_8_Pd_0.7_ ribbons calcined at different temperatures, and the average and variance of these data.

| **Calcination temperature (°C)** | **S_BET_** **(m^2^g^-1^)** | | | | **D_p_ (nm)** | | | | **V_p_ (cm^3^g^-1^)** | | | |
| --- | --- | --- | --- | --- | --- | --- | --- | --- | --- | --- | --- | --- |
|  | **1^st^** | **2^nd^** | **‾S** | **σ(S)** | **1^st^** | **2^nd^** | **‾D** | **σ(D)** | **1^st^** | **2^nd^** | **‾V** | **σ(V)** |
| **Dealloyed ribbons** | **80** | **76** | **78.5** | **2** | **13.66** | **14.27** | **13.97** | **0.305** | **0.267** | **0.283** | **0.275** | **0.0080** |
| **Calcined at 300 °C** | **101** | **98** | **99.5** | **1.5** | **12.02** | **12.36** | **12.19** | **0.17** | **0.336** | **0.332** | **0.334** | **0.0020** |
| **Calcined at 400 °C** | **102** | **109** | **105.5** | **3.5** | **13.72** | **13.12** | **13.42** | **0.3** | **0.362** | **0.364** | **0.363** | **0.0010** |
| **Calcined at 500 °C** | **85** | **94** | **89.5** | **4.5** | **12.73** | **12.93** | **12.83** | **0.1** | **0.304** | **0.332** | **0.318** | **0.0140** |
| **Calcined at 600 °C** | **84** | **90** | **87** | **3** | **13.21** | **13.82** | **13.52** | **0.305** | **0.317** | **0.324** | **0.321** | **0.0035** |

Table S4 Ratios of Ce, Pd, and O in different states for different catalysts as obtained from XPS results, and the average and variance of these data.

| Catalysts | Ce^3+^/(Ce^3+^+Ce^4+^)  (%) | | | | Pd^2+^/(Pd^0^+Pd^2+^)  (%) | | | | O_sur_/(O_lat_+O_sur_+O_H2O_)  (%) | | | |
| --- | --- | --- | --- | --- | --- | --- | --- | --- | --- | --- | --- | --- |
|  | **1^st^** | **2^nd^** | **‾Ce^3+^** | **σ(Ce^3+^)** | **1^st^** | **2^nd^** | **‾Pd^2+^** | **σ(Pd^2+^)** | **1^st^** | **2^nd^** | **‾O_sur_** | **σ(O_sur_)** |
| Calcined dealloyed Al_92_Ce_8_ | **14.27** | **15.12** | **14.70** | **0.425** | **/** | **/** | **/** | **/** | **/** | **/** | **/** | **/** |
| Calcined dealloyed Al_91.3_Ce_8_Pd_0.7_ | **23.33** | **23.17** | **23.25** | **0.080** | **91.25** | **95.24** | **93.25** | **1.995** | **29.3** | **31.0** | **30.15** | **0.850** |
| Dealloyed Al_91.3_Ce_8_Pd_0.7_ | **21.15** | **22.38** | **21.77** | **0.615** | **6.45** | **7.25** | **6.85** | **0.400** | **16.2** | **17.9** | **17.05** | **0.850** |

It can be seen from the data of these repeated experiments in Fig. S7~Fig. S10 and Table S3~Table S4 that the PdO/CeO_2_ catalyst prepared by combining dealloying with calcination has excellent reproducibility, which is very suitable for large-scale industrial production and practical applications.

**References**

[1] R. Burch, F.J. Urbano, P.K. Loader (1995) Methane combustion over palladium catalysts: The effect of carbon dioxide and water on activity. Appl. Catal. A: Gen. 123:173-184.

[2] S. Xie, Y. Liu, J. Deng, S. Zang, Z. Zhang, H. Arandiyan, H. Dai (2017) Efficient Removal of Methane over Cobalt-Monoxide-Doped AuPd Nanocatalysts. Environ. Sci. Technol. 51:2271-2279.

[3] M. Monai, T. Montini, C. Chen, E. Fonda, R.J. Gorte, P. Fornasiero (2015) Methane Catalytic Combustion over Hierarchical Pd@CeO_2_/Si-Al_2_O_3_: Effect of the Presence of Water. Chemcatchem. 7: 2038-2046.

[4] Y. Dai, V. Pavan Kumar, C. Zhu, M.J. MacLachlan, K.J. Smith, M.O. Wolf (2018) Mesoporous Silica-Supported Nanostructured PdO/CeO_2_ Catalysts for Low-Temperature Methane Oxidation. ACS Appl. Mater. Inter. 10:477-487.
